# Beta‐Cell Tipe1 Orchestrates Insulin Secretion and Cell Proliferation by Promoting Gαs/cAMP Signaling via USP5

**DOI:** 10.1002/advs.202304940

**Published:** 2024-02-28

**Authors:** Lu Ding, Yang Sun, Yan Liang, Jie Zhang, Zhendong Fu, Caiyue Ren, Pengfei Li, Wen Liu, Rong Xiao, Hao Wang, Zhaoying Zhang, Xuetian Yue, Chunyang Li, Zhuanchang Wu, Yuemin Feng, Xiaohong Liang, Chunhong Ma, Lifen Gao

**Affiliations:** ^1^ Key Laboratory for Experimental Teratology of Ministry of Education Shandong Key Laboratory of Infection and Immunity and Department of Immunology School of Basic Medical Sciences Cheeloo College of Medicine Shandong University Jinan Shandong 250012 P. R. China; ^2^ Department of Endocrinology Yucheng People's Hospital Dezhou Shandong 251200 P. R. China; ^3^ Key Laboratory for Experimental Teratology of Ministry of Education and Department of Cell Biology School of Basic Medical Sciences Cheeloo College of Medicine Shandong University Jinan Shandong 250012 P. R. China; ^4^ Key Laboratory for Experimental Teratology of Ministry of Education and Department of Histology and Embryology School of Basic Medical Sciences Cheeloo College of Medicine Shandong University Jinan Shandong 250012 P. R. China; ^5^ Department of Gastroenterology ShengLi Hospital of Shandong First Medical University Jinan Shandong 250012 P. R. China

**Keywords:** islet β cell, Tipe1, Gαs ubiquitination, deubiquitinase USP5, diabetes

## Abstract

Inadequate β‐cell mass and insulin secretion are essential for the development of type 2 diabetes (T2D). TNF‐α‐induced protein 8‐like 1 (Tipe1) plays a crucial role in multiple diseases, however, a specific role in T2D pathogenesis remains largely unexplored. Herein, Tipe1 as a key regulator in T2D, contributing to the maintenance of β cell homeostasis is identified. The results show that the β‐cell‐specific knockout of Tipe1 (termed *Ins2‐Tipe1BKO*) aggravated diabetic phenotypes in *db/db* mice or in mice with high‐fat diet‐induced diabetes. Notably, Tipe1 improves β cell mass and function, a process that depends on Gαs, the α subunit of the G‐stimulating protein. Mechanistically, Tipe1 inhibited the K48‐linked ubiquitination degradation of Gαs by recruiting the deubiquitinase USP5. Consequently, Gαs or cAMP agonists almost completely restored the dysfunction of β cells observed in *Ins2‐Tipe1BKO* mice. The findings characterize Tipe1 as a regulator of β cell function through the Gαs/cAMP pathway, suggesting that Tipe1 may emerge as a novel target for T2D intervention.

## Graphical abstract

Islet β‐cell homeostasis is essential for diabetes pathogenesis. TNF‐α‐induced protein 8‐like 1 (Tipe1) is highly expressed in β cells and inhibits diabetes progression. Mechanistically, Tipe1 inhibits the K48‐linked ubiquitination degradation of Gαs to improve cAMP/PKA/CREB pathway by recruiting the deubiquitinase USP5, thereby maintaining β cell homeostasis. This work provides a promising β cell target for diabetes intervention in clinical.

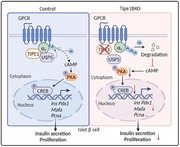

## 1 Introduction

Pancreatic β cells, the predominant cells in the islets of Langerhans,^[^ ^1^ ^]^ play a crucial role in maintaining insulin secretion and glucose homeostasis.^[^ ^2^, ^3^ ^]^ The pathogenesis of diabetes is mainly attributed to an inadequate supply of functional β cells.^[^ ^4^, ^5^ ^]^ Recent studies indicate that patients with type 1 or type 2 diabetes mellitus (T2D) possess significantly fewer islet β‐cells compared to healthy individuals.^[^ ^3^, ^6^ ^]^ It is widely known that changes in β cell mass and function are essential for the metabolic status and prognosis of T2D.^[^ ^5^ ^]^ Moreover, evidence has shown that key molecules implicated in regulating β‐cell mass and insulin secretion have been identified as promising candidate targets for T2D therapy,^[^ ^7^, ^8^, ^9^, ^10^ ^]^ underscoring the urgent need for the identification of novel targets for T2D interventions.

The tumor necrosis factor (TNF)‐α‐induced protein 8‐like 1 (Tipe1) belongs to the TNF‐α‐induced protein 8 (TNFAIP8) superfamily, and the family members have fairly high sequence homology.^[^ ^11^, ^12^ ^]^ Previous evidence has suggested that Tipe1 is involved in cell proliferation,^[^ ^13^, ^14^ ^]^ immunomodulation,^[^ ^15^, ^16^ ^]^ carcinogenesis^[^ ^17^, ^18^ ^]^ and diabetes‐related diseases.^[^ ^19^ ^]^ Although Tipe1 is highly expressed in the islets of normal mice,^[^ ^11^ ^]^ the roles of Tipe1 in β cell functions and the development of diabetes remain largely uncharacterized.

Pancreatic β cells, which are in charge of insulin secretion, play a pivotal role in determining the outcome of T2D. Insulin secretion‐related genes are regulated by several transcription factors, such as *MafA*, *Pdx1*, *Pax6*.^[^ ^2^, ^20^, ^21^, ^22^ ^]^ Emerging evidence suggests that these genes are indispensable for the maintenance of insulin secretion in β cells.^[^ ^8^, ^9^, ^23^ ^]^ Moreover, cyclic AMP (cAMP), acting as the downstream effectors has been found to enhance insulin biosynthesis, and secretion, and maintain β cell mass.^[^ ^24^ ^]^ The classical pathway of cAMP generation involves activation of transmembrane adenylate cyclase by G protein‐coupled receptors (GPCRs).^[^ ^25^ ^] ^The *Gnas* gene encodes the α subunit of G stimulating protein (Gαs), which acts as a molecular switch to transmit the GPCRs signals, controlling cell physiological and pathological processes by modulating cAMP production.^[^ ^26^, ^27^ ^]^ Several studies have revealed the importance of Gαs/cAMP pathway in regulating islet β cell function. As reported, β‐cell specific Gαs deficiency leads to insulin‐deficient diabetes, reduced glucose‐stimulated insulin secretion, and decreased β cell proliferation, which contributes to the progressive decline of β cell mass.^[^ ^28^, ^29^, ^30^ ^]^ These studies indicate that key signal transducers involved in the cAMP signaling pathway are promising targets for T2D intervention. Recently, it is reported that IL‐6 induces Gαs acetylation at K28, mediating its association with STAT3 and enhancing STAT3 activation.^[^ ^31^ ^]^ However, the post‐translational modification of Gαs protein involved in maintaining β cell homeostasis requires further investigation.

In the current study, we have, for the first time, confirmed the crucial role of Tipe1 in maintaining β cell mass and insulin secretion through modulating Gαs stability. Our work revealed that the mice harboring *insulin2* (*Ins2*)‐*Tipe1BKO* had aggravated diabetic phenotypes in diabetic mice. Mechanistically, Tipe1 inhibits the K48‐linked ubiquitination degradation of Gαs through the deubiquitinase USP5. This study identifies Tipe1 as an important regulator in β cell proliferation and function via Gαs/cAMP pathways, which might represent a novel therapeutic target for T2D intervention.

## 2 Results

### 2.1 High Expression of Tipe1 in Pancreatic Islet β Cells is Positively Correlated with the Levels of Insulin Related Genes

To explore the potential role of Tipe1 in regulating the function of pancreatic β cells, the distribution of Tipe1 expression in the pancreas was first identified. Immunohistochemical (IHC) staining, confirmed high expression of Tipe1 in human pancreatic islets (Figure S1A, Supporting Information). To further clarify the cell type expressing Tipe1, we used multiplexed immunofluorescence staining for Tipe1, insulin (β cell), glucagon (α cell), and somatostatin (δ cells) in human islets. Intriguingly, Tipe1 was found to be highly enriched in β cells, compared to α and δ cells (Figure S1B–D, Supporting Information). Consistently, high expression of Tipe1 was detected in islets of wide‐type (WT) mice and β cells (Figure S1E–G, Supporting Information).

To determine whether Tipe1 is involved in the regulation of insulin expression under physiological conditions, we measured its expression in the islets following dietary changes (Figure S2A, Supporting Information). The mRNA level of *Tipe1* in islets was reduced during fasting and was restored after refeeding, which was similar to the changes in insulin‐related genes (*Pdx1*, *MafA*, *Ins1*) (Figure S2B, Supporting Information) as well as the blood glucose and serum insulin levels (Figure S2C–E, Supporting Information). The Gene Expression Profiling Interactive Analysis (GEPIA) database (http://gepia.cancer‐pku.cn/)^[^ ^32^ ^]^ was used to evaluate the potential role of Tipe1 in healthy human pancreatic tissues, and the data showed a positive correlation between Tipe1 and proliferation‐related (*Pcna*, *Akt*, *Myc*, *Ccnd1*) or insulin‐related genes (*Pdx1*, *MafA*, *Pax6*, *Sla2a2*, *Kcnj11*) (Figure S2F). Collectively, these findings suggest that the expression of Tipe1 is positively correlated with the expression of key insulin‐related genes expression under physiological conditions, which further suggests the possible role of Tipe1 in the regulation of β cell function.

### 2.2 Tipe1 Knockdown in β Cells Causes Severe Diabetic Phenotypes in Mice

To assess the role of Tipe1 in β cells, we crossed *Tipe1‐floxed* (*Tipe1^f/f^*) mice with rat insulin II promoter (*Ins2*)‐Cre (commonly called *RIP‐Cre*) mice,^[^ ^8^, ^33^ ^]^ to generate β cell‐specific *Tipe1* deficiency mice, i.e., *Ins2^Cre+/−^Tipe1^f/f^* mice (hereafter referred to as *Ins2‐Tipe1BKO*) (Figure S3A,B, Supporting Information). Evidence shows that Ins2 is expressed in both the pancreatic islets and the hypothalamus.^[^ ^34^, ^35^ ^]^ However, we observed an extremely low protein level of Tipe1 in the hypothalamus compared to that in the islets (Figure S3C, Supporting Information). The expression of Tipe1 was markedly decreased in the islets of *Ins2‐Tipe1BKO* mice compared to that in control littermates (Figure S3C–F, Supporting Information). Immunofluorescence (IF) staining displayed that Tipe1 was significantly reduced in islet β cells of *Ins2‐Tipe1BKO* mice (Figure S3G, Supporting Information). When comparing *Ins2‐Tipe1BKO* mice to *Ins2‐Cre* mice, no significant differences were observed in body weight and random glucose levels (Figure S4A–C, Supporting Information); however, fasting blood glucose tended to be higher in *Ins2‐Tipe1BKO* mice (Figure S4D, Supporting Information). Surprisingly, 8–12‐week‐old male *Ins2‐Tipe1BKO* mice showed impaired glucose tolerance compared with *Ins2‐Cre* mice (Figure S4E, Supporting Information). Consistent with this, the insulin responses were markedly impaired in *Ins2‐Tipe1BKO* mice compared with the control littermates (Figure S4F, Supporting Information), although the insulin tolerance test (ITT) results showed high insulin sensitivity in *Ins2‐Tipe1BKO* mice (Figure S4G, Supporting Information). A significant decrease in islet area ratio and islet β cell mass was observed in *Ins2‐Tipe1BKO* mice (Figure S4H,I, Supporting Information). Collectively, these findings suggest that Tipe1 deficiency in β cells impairs glucose homeostasis.

**Figure 1:**
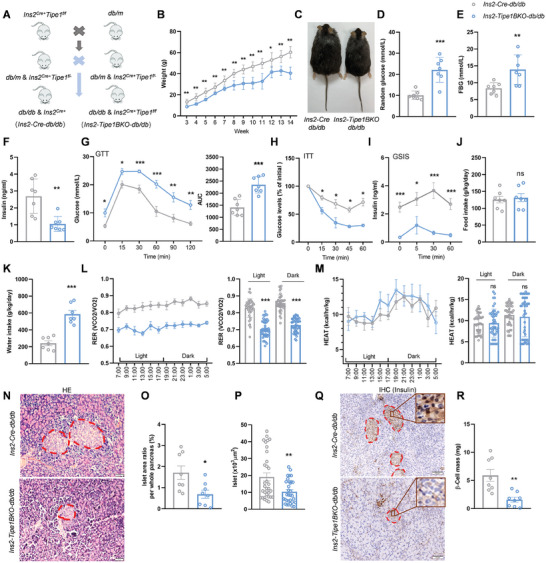
Tipe1 knockdown in β cells causes severe diabetic phenotypes in *db/db* mice. A) Strategy of islet β cell‐specific *Tipe1*‐knockout *db/db* mice. B) Growth curves of male *Ins2‐Tipe1BKO‐db/db* mice and *Ins2‐Cre‐db/db* mice. (*n* = 6 mice/group). C) Photograph of 14‐week‐old *Ins2‐Tipe1BKO‐db/db* mice and *Ins2‐Cre‐db/db* mice. D,E) Random blood and FBG levels were detected in 12‐week‐old male *Ins2‐Tipe1BKO‐db/db* mice and *Ins2‐Cre‐db/db* mice. (*n* = 7 mice/group). F) Fasting insulin levels were detected in 12‐week‐old male *Ins2‐Tipe1BKO‐db/db* mice and *Ins2‐Cre‐db/db* mice. (*n* = 7 mice/group). G) Plasma glucose levels during the glucose tolerance test for 12 weeks mice. (*n* = 7 mice/group). H) Plasma glucose levels during the ITT‐test for 12 weeks mice. (*n* = 3 mice/group). I) Blood insulin levels during GSIS‐test for 12 weeks mice. (*n* = 7 mice/group). J,K) Daily food and drink intake of *Ins2‐Cre‐db/db* and *Ins2‐Tipe1BKO‐db/db* mice. Mice were put in individually into metabolic cages. The food and drink intake were measured by a built‐in detector. (*n* = 7 mice/group). L,M) The RER and heat production of *Ins2‐Cre‐db/db* and *Ins2‐Tipe1BKO‐db/db* mice. Mice were put in individually into the ametabolic cage. The VO_2_, VCO_2,_ and oxygen consumed were measured by a built‐in detector. The RER was calculated by dividing VCO_2_/VO_2_. (*n* = 7 mice/group). N‐P) H&E staining was performed in pancreatic tissues of *Ins2‐Cre‐db/db* and *Ins2‐Tipe1BKO‐db/db* mice, followed by measurements of the islet area/pancreatic area ratio and islet area. (*n* = 8 mice/group). Q,R) IHC staining was used to detect the protein level of insulin in pancreatic sections, followed by measurements of β‐cell mass. (*n* = 8 mice/group). Data are presented as the mean ± SEM. Data were statistically analyzed by Student's *t*‐test. ^*^p < 0.05, ^**^p < 0.01, ^***^p < 0.001, ns indicates no significant difference.

The role of Tipe1 in islet β cells under T2D conditions was further investigated in Tipe1 knockout *db/db* mice. We bred *Ins2‐Tipe1BKO* (*Ins2^Cre+/−^Tipe1^f/f^*) mice with *db/m* mice, generating *db/db* mice with conditional Tipe1 knockout in β cells, i.e., mice hereafter referred to as *Ins2‐Tipe1BKO‐db/db* (**Figure** **1**A; Figure S3B, Supporting Information). Surprisingly, *Ins2‐Tipe1BKO‐db/db* mice showed significantly reduced growth at 3 weeks, whereas *Ins2‐Cre‐db/db* mice grew normally (Figure 1B,C). Tipe1 deletion in *db/db* mice led to higher random and fasting blood glucose levels (Figure 1D,E), lower fasting insulin levels (Figure 1F), and severe glucose intolerance compared to *Ins2‐Cre‐db/db* mice (Figure 1G). However, in *Ins2‐Tipe1BKO‐db/db* mice, the impaired glucose tolerance was not associated with insulin resistance, and markedly increased insulin sensitivity was observed based on their markedly acute hypoglycemic response to insulin (Figure 1H; Figure S5A, Supporting Information), with improved insulin signaling in skeletal muscle and adipose tissues (Figure S5B, Supporting Information). Glucose intolerance was accompanied by significantly reduced plasma insulin levels during the intraperitoneal glucose tolerance test, confirming the lack of insulin secretion in *Ins2‐Tipe1BKO‐db/db* mice (Figure 1I).

Evidence shows that people with prediabetes or diabetes are characterized by a reduced respiration exchange rate (RER) and metabolic inflexibility.^[^ ^36^, ^37^ ^]^ To investigate the reason why *Ins2‐Tipe1BKO‐db/db* mice have higher blood glucose levels and lesser body weights than *db/db* mice, we examined the food intake, drink intake, and energy expenditure using metabolic cages. Compared to *Ins2‐Cre‐db/db* mice, the food intake of *Ins2‐Tipe1BKO‐db/db* mice was not different; however, their water intake was greatly increased (Figure 1J,K). The RER of *Ins2‐Tipe1BKO‐db/db* mice significantly decreased, indicating that these mice had impaired glucose utilization and fat consumption, resulting in hyperglycemia and lower body weight compared to *Ins2‐Cre‐db/db* mice (Figure 1L). However, no significant alterations were observed in heat production in *Ins2‐Tipe1BKO‐db/db* mice (Figure 1M). Next, we evaluated changes in the islets of *Ins2‐Tipe1BKO‐db/db* mice and observed a significant decrease in the islet area ratio and islet area (Figure 1N–P). Consistent with this finding, the β cell mass dropped dramatically in *Ins2‐Tipe1BKO‐db/db* mice (Figure 1Q,R). Altogether, these findings suggest that Tipe1 deficiency in β cells of *db/db* mice accelerates β cell loss and insulin insufficiency under T2D conditions.

Evidence suggests that long‐term exposure to a high‐fat diet (HFD) can induce T2D models, leading to β cell decompensation by increasing metabolic stress.^[^ ^38^ ^]^ Thus, we identified the role of Tipe1 in regulating the reserve and function of β cells. In this study, we bred *Tipe1^f/f^* mice with pancreatic and duodenal homeobox 1 (*Pdx1*) or *Ins2‐Cre* transgenic mice to further characterize the regulatory role of Tipe1 in islets (Figure S6A–C, Supporting Information). Consistently, *Pdx1* or *Ins2‐Tipe1BKO* mice exhibited lower body weight gain (Figures S4J,K,S6D,E, Supporting Information), leading to higher FBG levels (Figure S6F, Supporting Information) and lower fasting insulin levels (Figure S6G, Supporting Information), worse glucose tolerance and reduced insulin secretion under HFD conditions (Figure S4L,M; Figure S6H,I, Supporting Information). In addition, these mice showed markedly increased insulin sensitivity based on their greater acute hypoglycemic response to insulin (Figure S4N; Figure S6J,K, Supporting Information). Meanwhile, a significant decrease in islet area and β cell mass was observed in *Pdx1‐Tipe1BKO* mice with HFD (Figure S6L,M, Supporting Information). Thus, Tipe1 loss reduces the compensatory proliferation of β cells under HFD stimulation, resulting in inadequate β cell mass.

### 2.3 Tipe1 Promotes β Cell Proliferation and Insulin Secretion

**Figure 2:**
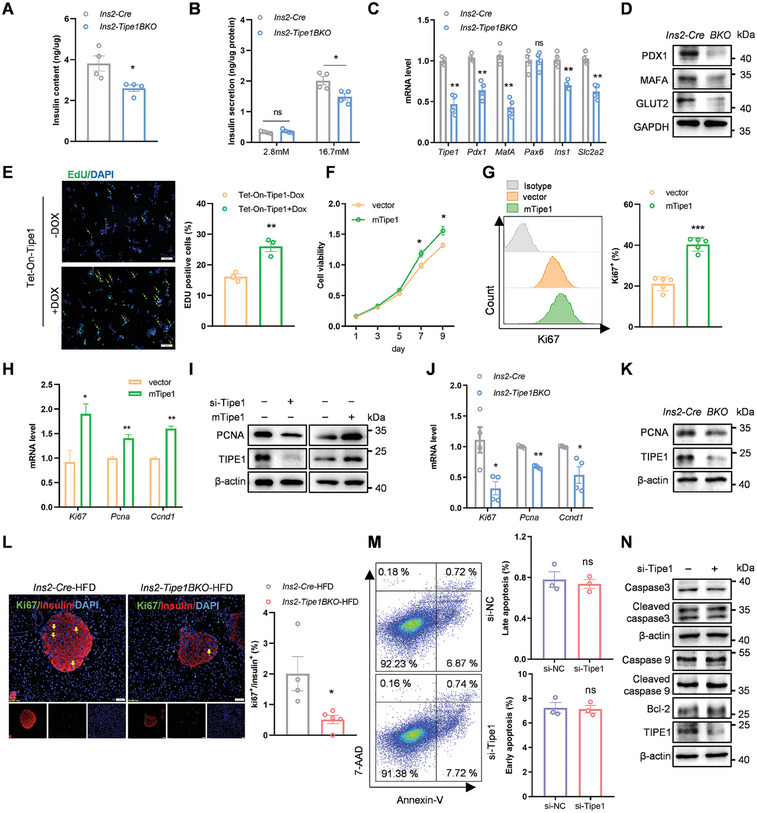
Tipe1 promotes β cell proliferation and insulin secretion in a cell‐autonomous manner. A) Pancreatic islets from 8‐week‐old *Ins2‐Cre* and *Ins2‐Tipe1BKO* mice were used to detect intracellular insulin levels. (*n = *4 mice/group). B) Pancreatic islets were treated with 2.8 or 16.7 mM glucose in an in vitro setting, and the insulin levels in the culture supernatants were determined by ELISA. (*n = *5 mice/group). C,D) Expression of the indicated genes in islets of *Ins2‐Cre* and *Ins2‐Tipe1BKO* mice was measured by qRT‐PCR or Western blotting. mRNA levels were normalized to *β‐actin* mRNA. (*n = *3 mice/group). E) MIN6 cells were infected with Tet‐On‐Tipe1 (‐DOX) or Tet‐On‐Tipe1 (+DOX) lentiviruses and subjected to EdU incorporation assays. The new generation cells were detected via EdU (green). DAPI stained nuclei blue. Merged view of EdU (green) and DAPI (blue) showing the overlap. Scale bar, 50 µm. (*n = *3). F) MIN6 cells were overexpressed with Tipe1 and cell viability was measured using the Cell Counting Kit‐8. (*n = *4). G) MIN6 cell proliferation was measured by flow cytometry. Flow cytometry histograms showing the level of Ki67 in MIN6 cell lines infected with vector or Tipe1‐overexpressed lentiviruses. (*n = *5). H) MIN6 cells were infected with Pultra‐NC or Pultra‐Tipe1 lentiviruses to overexpress Tipe1. Expression of the indicated genes in Min6 cells was measured by qRT‐PCR. mRNA levels were normalized to *β‐actin* mRNA. (*n = *3). I) MIN6 cells were silenced for Tipe1 expression or overexpressed with Tipe1 for 72 h. Expression of the PCNA in MIN6 cells was measured by Western blotting. J) Expression of the indicated genes in islets of *Ins2‐Cre* and *Ins2‐Tipe1BKO* mice was measured by qRT‐PCR. mRNA levels were normalized to *β‐actin* mRNA. (*n = *4 mice/group). K) Expression of PCNA in the islets of *Ins2‐Cre* and *Ins2‐Tipe1BKO mice* was measured by Western blotting. L) IF staining of Ki67. Ki67‐positive cells in islets were normalized to total insulin‐positive cells in the same area. Scale bar, 50 µm. (*n = *4 mice/group). M) Assessment of apoptosis in *Tipe1* silenced MIN6 cells analyzed by Annexin‐V and 7‐AAD by flow cytometry. (*n = *3). N) Expression of the apoptosis‐related genes in *Tipe1* silenced MIN6 cells was measured by Western blotting. Data are presented as the mean ± SEM. ^*^p < 0.05, ^**^p < 0.01, ^***^p < 0.001, ns (no significant difference) by Student's *t*‐test.

The aforementioned data confirmed that Tipe1 deletion caused β cell loss and decreased insulin secretion. To better understand the general effects of Tipe1 loss in β cells, we used isolated islets and MIN6 β‐cell‐line to examine whether Tipe1 could regulate β cell function in a cell‐autonomous manner. Primary islets were isolated from *Ins2‐Tipe1BKO* and *Ins2‐Cre* mice and subsequently treated with glucose concentrations of 2.8 or 16.7 mM in an in vitro setting. The results showed that intracellular insulin was decreased within the islets in *Ins2‐Tipe1BKO* mice. Furthermore, when islets were stimulated with 16.7 mM glucose, insulin secretion was significantly reduced in *Ins2‐Tipe1BKO* mice compared with that in *Ins2‐Cre* mice (**Figure** **2**A,B). Additionally, the intracellular insulin levels were significantly reduced in the islets of *Ins2‐Tipe1BKO‐db/db* mice (Figure S7A, Supporting Information). Consistently, the expression of insulin‐related genes was reduced in the islets of *Ins2‐Tipe1BKO* mice compared to that in *Ins2‐Cre* mice (Figure 2C,D). Next, we knocked down or increased Tipe1 expression in MIN6 cells. As expected, proliferation decreased in MIN6 cells with Tipe1 knockdown and increased with Tipe1 overexpression (Figure 2E–I). We isolated primary islets from *Ins2‐Tipe1BKO* mice and control littermates to assess islet proliferation. As expected, proliferation‐related markers were decreased in *Ins2‐Tipe1BKO* islets compared to those in *Ins2‐Cre* mice (Figure 2J,K). Evidence shows that prolonged (3–6 months) exposure to high lipids is recommended to increase β cell proliferation.^[^ ^38^, ^39^ ^]^ IF staining of insulin and the proliferation marker Ki‐67 (Ki67) revealed that β‐cell proliferation was significantly reduced in *Ins2‐Tipe1BKO*‐HFD and *Ins2‐Tipe1BKO‐db/db* mice compared with the control littermates (Figure 2L; Figure S7B, Supporting Information). We investigated whether Tipe1 knockdown was associated with cell apoptosis. Apoptosis assessed by annexin‐V and 7‐AAD staining revealed no differences in the percentage of apoptotic cells in Tipe1 silenced MIN6 cells compared to the negative control (Figure 2M). Likewise, apoptosis‐related gene expression in Tipe1 silenced MIN6 cells showed no significant differences compared to the control group (Figure 2N).

To assess the function of Tipe1 in islet β cells, an adenovirus overexpressing *Tipe1* was included in *Tipe1* silenced MIN6 cells and the islets of *Ins2‐Tipe1BKO* mice. MIN6 cells were infected with either Ad‐Con or Ad‐Tipe1, in which Ad‐Tipe1 is used to overexpress Tipe1(Figure S7C, Supporting Information). As expected, the proliferation of MIN6 cells was reversed in *Tipe1* overexpressing MIN6 cells with *Tipe1* deficiency (Figure S7D, Supporting Information). Moreover, high‐glucose‐stimulated insulin secretion was also reversed in the islets of *Ins2‐Tipe1BKO* mice overexpressing Tipe1 (Figure S7E, Supporting Information). The overexpression of Tipe1 consistently reversed the downregulation of insulin and proliferation‐related genes in the islets of *Ins2‐Tipe1BKO* mice (Figure S7F, Supporting Information). Overall, the above data demonstrate that Tipe1 promotes β cell proliferation and insulin secretion in a cell‐autonomous manner.

### 2.4 Tipe1 Maintains β Cell Function via Gαs/cAMP Pathway

**Figure 3:**
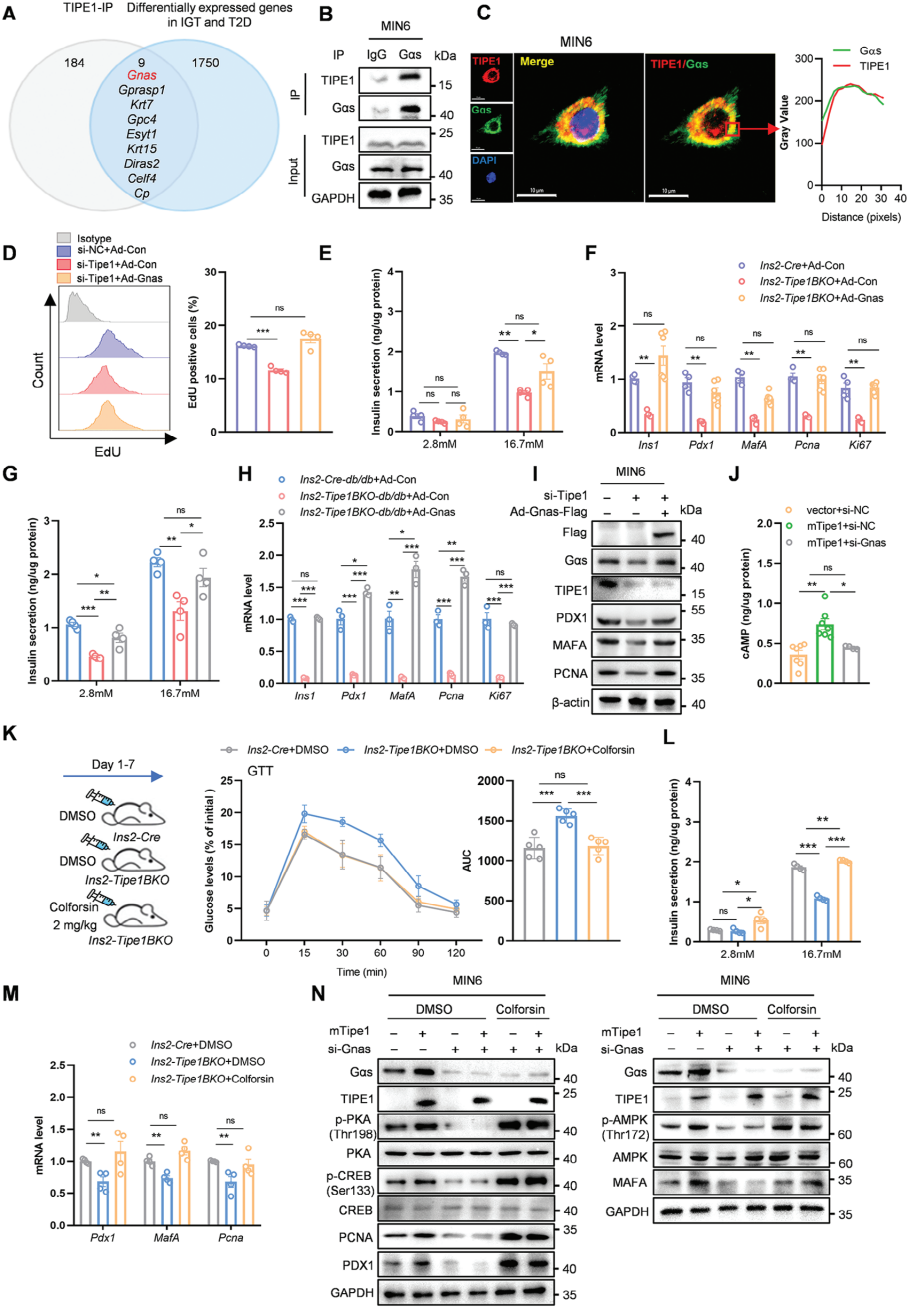
Tipe1 maintains β cell function via Gαs/cAMP pathway. A) Tipe1 interaction proteins in MIN6 cells and differentially expressed genes in human IGT and T2D (Hba1c > 6%, 0.2 < logFC <−0.2). B) Intracellular interaction between Tipe1 and Gαs. Endogenous TIPE1 was immunoprecipitated from MIN6 cell lysates and immunoblotted with antibodies against Gαs. C) MIN6 cells were subjected to IF staining with antibodies against TIPE1 (red), Gαs (green). Nuclei were stained with DAPI (blue). Scale bar, 10 µm. D) MIN6 cells silenced *Tipe1* for 24 h, then infected with either Ad‐Con or Ad‐Gnas for 48 h, were subjected to EdU incorporation assays by flow cytometry. Flow cytometry histograms showing the level of EdU in MIN6 cell lines. (*n = *4 for each group). E) Pancreatic islets from 8‐week‐old *Ins2‐Cre* and *Ins2‐Tipe1BKO* mice were treated with either Ad‐Con or Ad‐Gnas for 48 h, subsequently treated with 2.8 or 16.7 mM glucose in an in vitro setting. Insulin levels in the culture supernatants were determined by ELISA. (*n = *4 mice/group). F) Islets were treated as in E, and qRT‐PCR was performed to detect the indicated gene expression. mRNA levels were normalized to *β‐actin* mRNA (*n = *4 mice/group). G) Pancreatic islets from 12‐week‐old *Ins2‐Cre‐db/db* and *Ins2‐Tipe1BKO‐db/db* mice were treated with either Ad‐Con or Ad‐Gnas for 48 h, subsequently treated with 2.8 or 16.7 mM glucose in an in vitro setting. Insulin levels in the culture supernatants were determined by ELISA. H) Islets were treated as in G, and qRT‐PCR was performed to detect the indicated gene expression. mRNA levels were normalized to *β‐actin* mRNA. (*n = *4 mice/group). I) MIN6 cells were treated as in D, and protein levels of these genes were measured by Western blotting. J) MIN6 cells infected with either Pultra‐NC or Pultra‐Tipe1 lentiviruses for 24 h, then silenced *Gnas* for 48 h, and the intracellular cAMP level was detected by ELISA kit. K) 8‐week‐old male *Ins2‐Cre* or *Ins‐Tipe1BKO* mice were treated with intraperitoneal injection of cAMP agonist (Colforsin, 2 mg k^−1^g/day) or control (DMSO) for 1 week. GTT‐test in *Ins2‐Cre* and or *Ins2‐Tipe1BKO* mice. (*n = *5 mice/group). L) Pancreatic islets from 8‐week‐old *Ins2‐Cre* and *Ins2‐Tipe1BKO* mice were treated with cAMP agonist (Colforsin, 10 µM) for 48 h, subsequently treated with 2.8 or 16.7 mM glucose in an in vitro setting. Insulin levels in the culture supernatants were determined by ELISA. (*n = *4 mice/group). M) Islets were treated as in L, and qRT‐PCR was performed to detect the indicated gene expression. mRNA levels were normalized to *β‐actin* mRNA. (*n = *4 mice/group). N) MIN6 cells infected with either Pultra‐NC or Pultra‐Tipe1 lentiviruses and siRNA targeting *Gnas* for 48 h, and then treated with cAMP agonist (Colforsin, 10 µM) for 48 h. The protein levels of indicated genes were detected by Western blotting. Data are presented as the mean ± SEM. ^*^p < 0.05, ^**^p < 0.01, ^***^p < 0.001, ns (no significant difference) by Student's *t*‐test.

Next, we attempted to determine the molecular mechanism by which Tipe1 regulates islet β cells. Using immunoprecipitation (IP)/mass spectrometry, we identified a large number of potential Tipe1 interacting proteins in β cells; next a cluster analysis with reported, differently expressed genes in human IGT (impaired glucose tolerance) and T2D islets (GSE50398)^[^ ^40^ ^]^ was performed. As shown in **Figure** **3**A, the following genes were enriched in clusters: *Gnas*, *Gprasp1*, *Krt7*, *Gcp4*, *Esyt1*, *Krt15*, *Diras2*, *Celf4*, *Cp*; these may be the genes related to Tipe1 that regulate β cell function. The roles of *Gnas*, *Gprasp1*, *Krt7*, and *Gcp4* have been reported in glycolipid metabolism,^[^ ^41^, ^42^, ^43^, ^44^ ^]^ among which *Gnas* plays a key role in regulating β cell function and has been listed as a candidate gene for T2D pathogenesis.^[^ ^28^, ^29^, ^40^ ^]^ *Krt7* is a constituent of the keratin network in mouse islets; however, *Krt7* is not expressed in human pancreatic islets under basal conditions.^[^ ^45^ ^]^ Gcp4 is located in the membrane,^[^ ^44^ ^]^ indicating an indirect interaction and regulation between Tipe1 and Gcp4. We next determined the interaction of Tipe1 with Gαs (encoded by *Gnas*) or GΑSP1 (encoded by *Gprasp1*) (Figure 3B; Figure S8A, Supporting Information). Meanwhile, the Confocal assay further verified the co‐localization of Tipe1 and Gαs in MIN6 cells (Figure 3C). We used the islets of *Ins2‐Tipe1BKO* mice and Tipe1 silenced MIN6 cells to verify the changes in *Gprasp1* expression. However, no significant changes of *Gprasp1* were found (Figure S8B–D, Supporting Information). To determine the effect of *Gprasp1*, we used si‐Gprasp1 to treat MIN6 cells with Tipe1 overexpression, and the results showed no significant changes in key insulin‐related genes in MIN6 cells (Figure S8E, Supporting Information). To confirm whether Gαs is involved in the Tipe1‐mediated regulation of islet β cells, adenovirus overexpressing *Gnas* was included in Tipe1 silenced MIN6 cells and the islets of *Ins2‐Tipe1BKO* mice. As expected, the proliferation of MIN6 cells was reversed in *Gnas* overexpressing MIN6 cells with Tipe1 deficiency (Figure 3D; Figure S9A, Supporting Information). Moreover, high‐glucose‐stimulated insulin secretion was also reversed by ≈40%–80% in the *Gnas*‐overexpressed islets of *Ins2‐Tipe1BKO* and *Ins2‐Tipe1BKO‐db/db* mice (Figure 3E,G; Figure S9B,C, Supporting Information). Consistent with this finding, *Gnas* overexpression reversed the downregulation of insulin and proliferation‐related genes in islets of *Ins2‐Tipe1BKO* mice, islets of *Ins2‐Tipe1BKO‐db/db* mice, and Tipe1 silenced MIN6 cells (Figure 3F‐I). These data demonstrate that Gαs controls Tipe1‐mediated regulation on β cells.

Several studies have reported the importance of Gαs/cAMP pathway in islet β cell function.^[^ ^29^, ^30^ ^]^ cAMP is found to promote insulin biosynthesis, insulin secretion, and β cell mass.^[^ ^24^ ^]^ We detected significantly up‐regulated cAMP levels in Tipe1‐overexpressed MIN6 cells; however, this upregulated cAMP level was reversed after *Gnas* silencing (Figure 3J). Evidence shows that the increased intracellular cAMP and subsequent activation of protein kinase A (PKA) play important roles in β cell function.^[^ ^46^, ^47^ ^]^ We detected changes in p‐PKA and p‐CREB (downstream signaling of PKA^[^ ^48^ ^]^) levels and found that p‐PKA and p‐CREB levels were downregulated in the *si‐Tipe1* group and were reversed in *Gnas*‐overexpression group (Figure S9D, Supporting Information). To evaluate the role of cAMP in *Ins2‐Tipe1BKO* mice, the mice were treated by intraperitoneal injection of cAMP agonist (2 mg k^−1^g day^−1^) for 1 week, and the results showed a significant improvement in glucose tolerance (Figure 3K). Islets of *Ins2‐Tipe1BKO* mice were isolated and treated with cAMP agonists for 48 h, as expected, the insulin secretion was completely reversed by high‐glucose stimulation (Figure 3L). Correspondingly, treatment with a cAMP agonist reversed the downregulation of insulin and proliferation‐related genes in the islets of *Ins2‐Tipe1BKO* mice (Figure 3M). Finally, Tipe1 was overexpressed in MIN6 cells, *Gnas* was silenced and the cells were treated with cAMP agonists. This treatment led to a complete reversal of down‐regulated protein levels of key β cell genes, namely, p‐PKA, p‐CREB, and p‐AMPK (downstream signaling of cAMP and PKA) were completely reversed (Figure 3N; Figure S9E–G, Supporting Information). It is well known that glucagon‐like peptide‐1 receptor (GLP‐1R) activation enhances insulin secretion by increasing cAMP production in β cells, further demonstrating the important role of Gαs in this regard.^[^ ^30^, ^49^ ^]^ Islets of *Ins2‐Cre* and *Ins2‐Tipe1BKO* mice were treated with GLP‐1 agonist (exendin 4) for 48 h, and, as expected, a decrease in Gαs in *Ins2‐Tipe1BKO* mice affected GLP‐1‐dependent regulation of insulin secretion in β cells (Figure S9H, Supporting Information). Overall, these data indicate that Tipe1 works through Gαs/cAMP pathways to regulate β cell proliferation and insulin secretion.

### 2.5 Tipe1 Stabilizes Gαs by Repressing the K48‐Linked Ubiquitination Degradation

The above data reveal that Tipe1 interacts with Gαs in MIN6 cells (Figure 3B). To further explore the specific interaction domain of Tipe1 and Gαs, several assays were conducted. Co‐IP assays further confirmed the interaction between Tipe1 and Gαs in HEK293T cells (Figure S10A, Supporting Information). Meanwhile, confocal assay further verified the co‐localization of Tipe1 and Gαs both in HeLa cells and islets (Figure S10B,C, Supporting Information). The next step involved mapping the key domain responsible for the interaction of Tipe1 and Gαs. We first clarified the Gαs binding domain of Tipe1. For this purpose, Flag‐tagged Tipe1 truncate mutants were constructed and co‐transfected with HA‐tagged Gαs in HEK293 cells, followed by Co‐IP assays with indicated antibodies. The results showed that both the N‐terminal and C‐terminal of Tipe1 were required for physical interaction with Gαs (Figure S10D, Supporting Information). Concurrently, further efforts were also made to define the critical region of Gαs that interacts with Tipe1. As shown in Figure S10E (Supporting Information), both of the Gαs mutants could interact with Tipe1. Taken together, these aforementioned findings demonstrate that Tipe1 interacts with Gαs, with little domain specificity.

**Figure 4:**
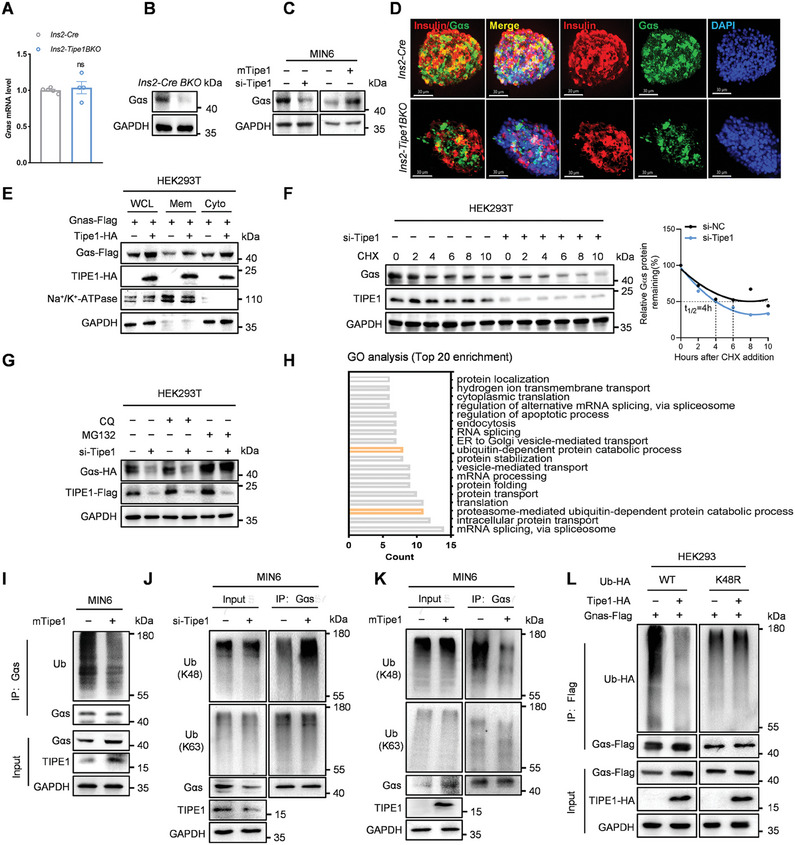
Tipe1 stabilizes Gαs by inhibiting K48‐linked ubiquitination. A,B) Gnas expression in islets of *Ins2‐Cre* and *Ins2‐Tipe1BKO* mice was measured by qRT‐PCR and Western blotting. mRNA levels were normalized to *β‐actin* mRNA. (n/*Ins2‐Cre* = 5; n/*Ins2‐Tipe1BKO* = 4). C) MIN6 cells were silenced Tipe1 or infected with Pultra‐NC (vector) or Pultra‐Tipe1 lentiviruses, and the expression of Gαs was detected by Western blotting. D) Isolated islets from *Ins2‐Cre* and *Ins2‐Tipe1BKO* mice were subjected to IF staining with antibodies against Insulin (red), Gαs (green). Nuclei were stained with DAPI (blue). A confocal assay was performed. Scale bar, 30 µm. E) HEK293T cells were transfected with human Tipe1‐HA and human‐Gnas‐Flag overexpression plasmid for 48 h. Membrane and cytoplasm fractions were isolated, and Gαs‐Flag levels of whole cell lysates (WCL), membrane (Mem), and cytoplasm (Cyto) were examined using Western blotting analysis. F) Cycloheximide (CHX) chase assay. HEK293T cells transfected with si‐*Tipe1* were treated with CHX (500 µg/ml) for the indicated time points. G) Western blotting analysis of HEK293T cells transfected with *Tipe1* siRNA and treated with Chloroquine (CQ, 10 µM) for 12 h or MG132 (20 µM) for 6 h. H) Proteins were identified as molecular interactors when using IP by Flag‐tagged Tipe1 and mass spectrometry (by GO function determination), and the top 20 GO clusters are displayed. I) Immunoblotting analysis of MIN6 cells transfected with mouse Tipe1 plasmid, followed by IP with anti‐Gαs, probed with anti‐Ub. J) MIN6 cells were overexpressed Tipe1, followed by IP with anti‐Gαs, and probed with anti‐K48‐Ub or K63‐Ub. K) MIN6 cells were silenced Tipe1, followed by IP with anti‐Gαs, and probed with anti‐K48‐Ub or K63‐Ub. L) HEK293T cells were transfected with human Tipe1‐HA, human‐Gnas‐Flag, Ub‐HA, or Ub‐K48R‐HA overexpression plasmid for 48 h, followed by IP with anti‐Flag, probed with anti‐HA. Data are presented as the mean ± SEM. ns (no significant difference) by Student's *t*‐test.

Notably, we detected a down‐regulated protein level of Gαs in the islets of *Ins2‐Tipe1BKO* mice and Tipe1 silenced MIN6 cells, on the contrary, the upregulated protein level of Gαs was detected in MIN6 cells overexpressing Tipe1 (**Figure** **4**A–C). In addition, IF staining showed remarkably reduced expression of Gαs in islets of *Ins2‐Tipe1BKO* mice (Figure 4D). To further confirm the effects of Tipe1 on Gαs protein expression, we overexpressed Tipe1 in HEK293T cells, and the results showed that ectopic Tipe1 increased Gαs protein abundance in the cytoplasm (Figure 4E). Consistently, following treatment with the protein synthesis inhibitor cycloheximide (CHX), Tipe1 deficiency reduced the half‐life of the Gαs protein in HEK293T cells (Figure 4F). On the contrary, Tipe1 overexpression increased the half‐life of the Gαs protein in HEK293T cells (Figure S11A, Supporting Information). Concurrently, treatment of HEK293T cells with proteasome inhibitor MG132, instead of autophagy inhibitor chloroquine (CQ), inhibited Gαs degradation and reversed the reduction in Gαs protein caused by Tipe1 deficiency (Figure 4G). Consistently, MG132 also abolished the Tipe1‐initiated accumulation of Gαs protein (Figure S11B, Supporting Information). All these data suggest that Tipe1 enhances Gαs abundance by blocking its proteasomal degradation.

To further elucidate the potential molecule that mediates the Tipe1‐initiated inhibition on Gαs protein degradation, a Gene Ontology analysis was performed using the potential Tipe1‐interacting partner obtained from mass spectrometry. The results showed that these proteins were involved in modulating RNA splicing, protein transport, and protein ubiquitination processes (Figure 4H). It has been reported that Tipe1 could inhibit the polyubiquitination of its binding proteins,^[^ ^50^, ^51^ ^]^ and that Gαs has an acetylation modification at K28,^[^ ^31^ ^]^ however, there is no evidence regarding Gαs ubiquitination modification. We then sought to determine whether Tipe1 could modulate the ubiquitination modification of Gαs. First, we detected the ubiquitination accumulation of endogenous Gαs in HEK293T and MIN6 cells. The results showed that the ubiquitination modification was detected in immunoprecipitated Gαs protein. Concurrently, Tipe1 overexpression inhibited ubiquitination accumulation on both endogenous and exogenous Gαs protein (Figure 4I; Figure S11C,D, Supporting Information), while Tipe1 knockdown promoted the ubiquitination of Gαs protein (Figure S11E, Supporting Information). Consistently, Tipe1 overexpression inhibited the K48‐linked ubiquitination of Gαs in MIN6 cells, while knockdown of Tipe1 reversed the aforementioned effects (Figure 4J–L). We then attempted to determine the ubiquitination sites of Gαs, using the following online database to map the potential ubiquitination sites on Gαs: http://bdmpub.biocuckoo.org/, http://iuucd.biocuckoo.org/, and http://gpsuber.biocuckoo.cn/. We generated a series of Gαs mutants containing only one lysine residue at various positions 8, 28, 53, 58, 88, 91, 96, 274, 300, 305, 307, or 338, or all lysine residues to arginine mutant (AKR). Immunoprecipitation assays showed predominant ubiquitination of Gαs at positions 8, 28, 96, or 307 (Figure S11F, Supporting Information), and overexpression of Tipe1 reduced the ubiquitination of Gαs at positions 8, 28, and 96, this indicates that the three lysine residues (K8, K28 and K96) of Gαs are responsible for Tipe1‐mediated polyubiquitination of Gαs (Figure S11G, Supporting Information). Collectively, our results demonstrate that Tipe1 stabilizes Gαs protein by inhibiting its K48‐linked ubiquitination and degradation.

### 2.6 Tipe1 Impedes K48 ‐Linked Ubiquitination of Gαs by Recruiting USP5

**Figure 5:**
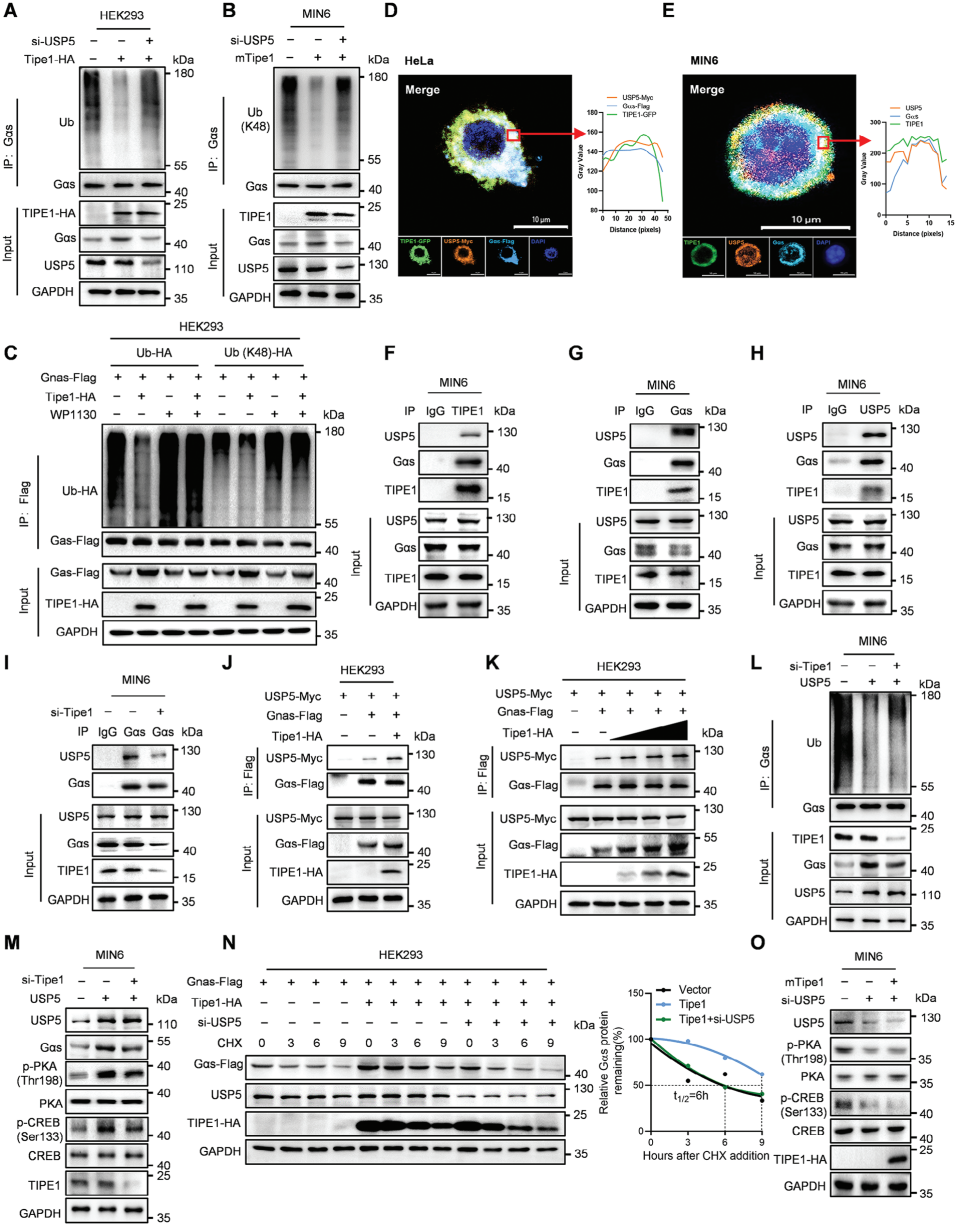
Tipe1 inhibits Gαs K48‐linked ubiquitination by USP5. A) Immunoblotting analysis of lysates from HEK293 cells silenced for USP5 but overexpressing Tipe1, followed by IP with anti‐Gαs, probed with anti‐Ub. B) MIN6 cells infected with Pultra‐NC or Pultra‐Tipe1 lentiviruses and silenced *USP5* for 48 h, followed by Co‐IP with anti‐Gαs, and probed with anti‐K48‐Ub. C) HEK293T cells were transfected with HA‐tagged Tipe1, Flag‐tagged Gnas, HA‐tagged Ub, or HA‐tagged K48‐linked ubiquitin (K48‐Ub) for 48 h, then treated with USP5 inhibitor (WP1130, 10 µM) for 6 h, followed by IP with anti‐Flag, and probed with anti‐HA. D) HeLa cells were transfected with Flag‐tagged Gnas, GFP‐tagged Tipe1, and Myc‐tagged USP5 for 48 h, then subjected to IF staining with antibodies against Gαs‐Flag (azure) and USP5‐Myc (orange), Nuclei were stained with DAPI (blue). A confocal assay was performed. Scale bar, 10 µm. E) MIN6 cells were subjected to IF staining with antibodies against TIPE1 (green), Gαs (azure), and USP5 (orange), Nuclei were stained with DAPI (blue). A confocal assay was performed. Scale bar, 10 µm. F‐H) Co‐IP with anti‐IgG, anti‐Tipe1, anti‐Gαs, or anti‐USP5, probed with anti‐Gαs, USP5 and Tipe1 in MIN6 cells. I) MIN6 cells transfected with *Tipe1* si‐RNA, followed by IP with anti‐IgG, anti‐Gαs, proved with anti‐USP5 and anti‐Gαs. J,K) HEK293 cells overexpressing Tipe1‐HA, Gnas‐Flag, and USP5‐Myc, followed by IP with anti‐Flag, proved with anti‐Flag and anti‐Myc. L) MIN6 cells were silenced *Tipe1* for 24 h, then infected with either pLVX‐Con or pLVX‐USP5 for 48 h, followed by IP with anti‐Gαs, probed with anti‐Ub. M) MIN6 cells were silenced *Tipe1* for 24 h, then infected with pLVX‐Con or pLVX‐USP5 for 48 h, and the protein levels of indicated genes were detected by Western blotting. N) HEK293 cells silenced for USP5 expression and transfected with Tipe1‐HA, Gnas‐Flag for 48 h, then treated with CHX (500 µg ml^−1^) for the indicated time points. O) MIN6 cells silenced *USP5* for 24 h, then infected with Ad‐Con or Ad‐*Tipe1* for 48 h, the protein levels of indicated genes were detected by Western blotting.

To elucidate the molecular mechanism by which Tipe1 modulates the ubiquitination degradation of Gαs protein, we focused on the captured ubiquitin‐associated proteins, which include several ubiquitinases (MAEA, WWP2) and deubiquitinases (USP9X, USP5) (Figure S11H, Supporting Information). To further identify the protein that interacts with Tipe1‐initiated regulation on Gαs ubiquitination, Co‐IP assays were used to verify the interaction of Tipe1 with the aforementioned ubiquitin‐associated proteins (Figure S11I,J, Supporting Information). The results showed that only the knockdown of USP5, rather than MAEA, WWP2, or USP9X, rescued the Tipe1‐induced inhibition on Gαs ubiquitination (**Figure** **5**A,B; Figure S11K–M, Supporting Information). Furthermore, when the USP5 inhibitor WP1130 was included, the results showed that WP1130 treatment almost completely reversed the Tipe1‐induced decrease in Gαs ubiquitination (Figure 5C). Interestingly, confocal assay further confirmed the exogenous and endogenous co‐localization of Tipe1, Gαs, and USP5 in HeLa and MIN6 cells (Figure 5D,E). Co‐IP assays also showed the protein interactions among Tipe1/Gαs/UPS5 in MIN6 cells (Figure 5F–H). Subsequently, we came to evaluate the effect of Tipe1 on the interaction of Gαs and USP5. Co‐IP assays showed that Tipe1 knockdown inhibited the interaction of USP5 and Gαs (Figure 5I), however, Tipe1 overexpression significantly promoted the interaction of USP5 and Gαs in a dose‐dependent manner (Figure 5J,K). Consistently, Tipe1 knockdown inhibited deubiquitylation of Gαs and reduced protein levels of Gαs, p‐PKA, p‐CREB in USP5 overexpressed MIN6 cells (Figure 5L,M). Moreover, we characterized whether USP5 was indispensable for Tipe1‐initiated stabilization of Gαs expression. Following treatment with CHX, the knockdown of USP5 almost completely rescued the Tipe1‐prolonged half‐life of Gαs in HEK293 cells (Figure 5N). Next, we explored how USP5 is involved in the regulation of Gαs/cAMP/PKA signaling downstream effectors. We silenced USP5 expression and overexpressed Tipe1 in HEK293 and MIN6 cells and found that Tipe1 overexpression did not reverse the protein levels of Gαs, p‐PKA, p‐CREB in USP5 silenced cells (Figure 5N,O). Taken together, these findings demonstrate that Tipe1 inhibits Gαs ubiquitination degradation via USP5.

### 2.7 Expression of Tipe1 and Gαs is Negatively Correlated with Fasting Glucose Levels in Human Islets

**Figure 6:**
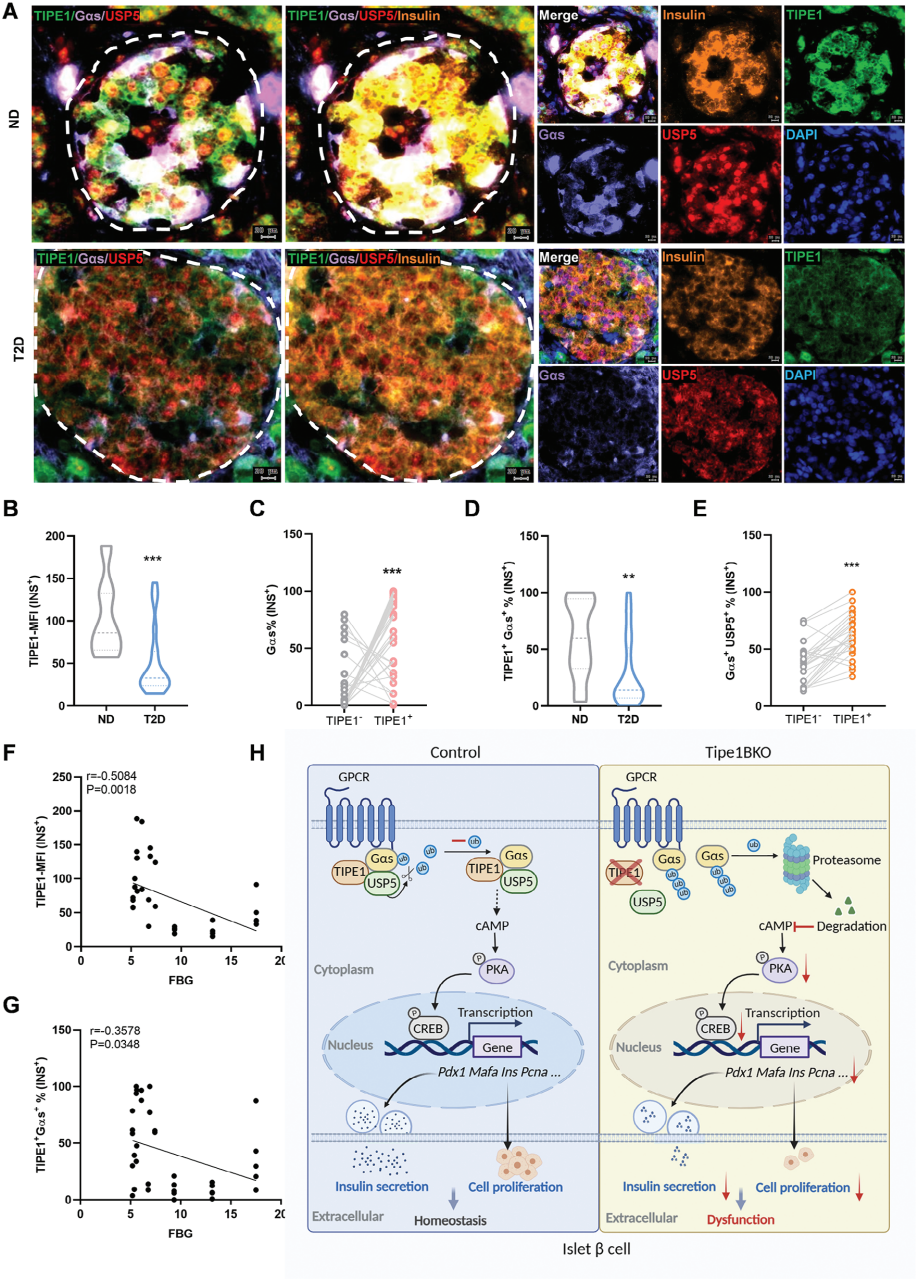
Negative correlation of blood glucose level with Tipe1 and Gαs expression in human islet β cells. A) Representative images for multiplexed immunofluorescence staining (TIPE1, green; Insulin, orange; USP5, red; Gαs, purple) in pancreatic paracancerous tissues from pancreatic cancer patients with and without T2D. B) The MFI for the expression of Tipe1 in human islet β cells of paracancerous tissues from pancreatic cancer patients with and without T2D. (n/ND = 6, n/T2D = 7). C) The frequency for Gαs positive cells in Tipe1 negative or positive islet β cells in human islet β cells of paracancerous tissues from pancreatic cancer patients. (n/Islet = 36). D) The frequency for Gαs and Tipe1 double‐positive cells in human islet β cells of pancreatic tissue of paracancerous from pancreatic cancer patients with and without diabetes. (n/ND = 6, n/T2D = 7). E) The frequency for Gαs and USP5 double‐positive cells in Tipe1 negative or positive islet β cells of pancreatic paracancerous tissues from pancreatic cancer patients. (n/Islet = 36). F) Correlation of fasting blood glucose with the MFI of Tipe1 expression in islet β cells of pancreatic paracancerous tissues from pancreatic cancer patients with and without T2D. (n/Islet = 36). G) Correlation of fasting blood glucose with the frequency of Gαs and Tipe1 double‐positive cells in islet β cells of pancreatic paracancerous tissues from pancreatic cancer patients with and without T2D. (n/Islet = 36). H) A schematic illustration showing a plausible mechanism underlying Tipe1 orchestrating islet β cell insulin secretion and proliferation by promoting Gαs/cAMP signaling via USP5. Data are presented as the mean ± SEM. ^**^p < 0.01, ^***^p < 0.001 by Student's *t*‐test.

To illustrate the clinical significance of Tipe1 in T2D, we used multiplexed immunofluorescence staining for Tipe1, insulin, Gαs, and USP5 in human islets of T2D patients and non‐diabetic (ND) individuals. Surprisingly, the expression of Tipe1 was reduced in human islet β cells of patients with T2D compared to ND individuals (**Figure** **6**A,B). We also found the expression of Tipe1 was reduced in mice islet β cells of *db/db* and HFD‐feeding mice compared to WT (Figure S12A,B, Supporting Information). Consistently, a GEO dataset (GSE50398) shows that the mRNA level of Tipe1 was decreased in the pancreatic islets of patients with T2D (Figure S12C, Supporting Information). Notably, Tipe1 was markedly reduced in the islets of 16–20‐week‐old *db/db* mice compared to *db/m* mice (Figure S12D, Supporting Information). Consistently, MIN6 cells exposed to high glucose (33.3 mM) for 48 h exhibited reduced Tipe1 expression (Figure S12E,F, Supporting Information). However, we did not characterize the key factors responsible for Tipe1 decrease in islet β cells under the high glucose microenvironment of T2D, and we did not elucidate potential mechanisms in this study, which requires further exploration.

The aforementioned results revealed that Tipe1 could stabilize Gαs (Figure 4F). Therefore, we analyzed the expression of Gαs in Tipe1‐positive and Tipe1‐negative islet β cells. Notably, Tipe1‐positive β cells expressed a higher proportion of Gαs in human and mouse islet β cells (Figure 6C; Figure S12G, Supporting Information). There was a significant reduction in the proportion of Tipe1 and Gαs double‐positive cells in the islet β cells of patients with T2D (Figure 6D). In addition, the multiplexed immunofluorescence staining in human islet tissues showed co‐expression among Tipe1, Gαs, and USP5 (Figure 6A). We analyzed the double‐positive proportion of Gαs and USP5 in Tipe1‐positive and Tipe1‐negative islet β cells. As anticipated, Tipe1‐positive β cells expressed a higher proportion of human islet β cells that were double‐positive for Gαs and USP5 (Figure 6E). As shown in Figure S2, the expression of Tipe1 correlated with the fasting status. Therefore, fasting blood glucose levels were used for further analysis (Table S1, Supporting Information). Notably, the expression level of Tipe1 was negatively correlated with fasting blood glucose in human islet β cells (Figure 6F). Furthermore, the proportion of Tipe1 and Gαs double positive cells was also negatively correlated with fasting blood glucose in human islet β cells (Figure 6G). In summary, we found a negative correlation between blood glucose levels and the expression of Tipe1 and Gαs in human islet β cells, further indicating that Tipe1 has a potential therapeutic role as a regulator of Gαs in diabetes mellitus.

## 3 Discussion

Herein, we report the novel roles of the Tipe1 gene in protecting islet β cell function in T2D. Emerging evidence has shown that Tipe1 is an important factor in tumorigenesis and immunology;^[^ ^13^, ^14^, ^15^, ^16^, ^17^ ^]^ however, its physiological functions are still poorly understood. Our study identifies Tipe1 in regulating β cell mass and insulin secretion by inhibiting Gαs K48‐linked polyubiquitination through deubiquitinases USP5 (Figure 6H).

Hepatocyte‐specific deletion of Tipe1 exacerbated diet‐induced hepatic steatosis and systemic metabolic disorders during nonalcoholic steatohepatitis pathogenesis.^[^ ^50^ ^]^ However, little is known regarding the roles of Tipe1 in diabetes mellitus. In this study, we found that Tipe1 deficiency in islet β cells led to pre‐diabetes phenotype, including glucose intolerance and impaired insulin secretion under physiological conditions (Figure S4, Supporting Information). Moreover, Tipe1 knockdown caused severe diabetes phenotypes in obese conditions (Figure 1; Figures S4 and S5, Supporting Information). Using IP/mass spectrometry, we identified a large number of potential Tipe1 interaction molecules in β cells, including Gαs, which is known to be the β cell regulator.

As shown in Figure S4A–D (Supporting Information), *Ins2‐Tipe1BKO* mice had normal body weight and blood glucose levels. However, *Ins2‐Tipe1BKO‐db/db* mice exhibited significant growth retardation and high blood glucose levels (Figure 1B–E). Emerging evidence shows that β cell‐specific Gαs deficiency leads to insulin‐deficient diabetes, causes high blood glucose, reduces glucose‐stimulated insulin secretion, and decreases β cell mass.^[^ ^28^, ^30^ ^]^ More importantly, Gαs deficiency in β cells causes small body size in mice,^[^ ^29^ ^]^ which is consistent with the phenotypes observed by us in *Ins2‐Tipe1BKO‐db/db* mice. Several studies report that growth hormone levels are reduced in patients with T2D owing to high blood glucose levels.^[^ ^52^, ^53^ ^]^ We detected fasting insulin and growth hormone levels in *Ins2‐Tipe1BKO‐db/db* and *Ins2‐Cre‐db/db* mice at 1 month of age and found that fasting insulin and growth hormone levels were significantly reduced in *Ins2‐Tipe1BKO‐db/db* mice (Data not show). In addition, the metabolic cage test showed that the RER of *Ins2‐Tipe1BKO‐db/db* mice was significantly decreased, indicating that *Ins2‐Tipe1BKO‐db/db* mice had impaired glucose utilization and fat consumption, resulting in hyperglycemia and lower body weight compared to *Ins2‐Cre‐db/db* mice (Figure 1L). As shown in Figure 1F, the glucose intolerance in *Ins2‐Tipe1BKO‐db/db* mice was not associated with insulin resistance, in fact, these mice had markedly increased in vivo insulin sensitivity based on their markedly greater acute hypoglycemic response to insulin. It seems that the increased insulin sensitivity often reflects the improved insulin signaling in skeletal muscle and adipose tissues.^[^ ^54^, ^55^ ^]^ Consistently, we also observed the improved insulin signaling in perirenal adipose tissue, epididymal adipose tissues, and skeletal muscles of *Ins2‐Tipe1BKO‐db/db* mice (Figure S5B, Supporting Information). The increased insulin sensitivity may be due to chronically low circulating insulin levels, which might be a feedback regulation caused by the secondary response to the reduced downregulation of insulin pathways.^[^ ^28^ ^]^ Several studies have reported the importance of Gαs/cAMP pathway in islet β cell functions.^[^ ^28^, ^29^, ^30^ ^]^ In our study, overexpression of Gαs or treatment with a cAMP agonist almost completely restored the dysfunction of β cells in *Ins2‐Tipe1BKO* mice (Figure 3E–I,K–N). Furthermore, given the beneficial effect of Tipe1 on β‐cell function, specifically overexpressing Tipe1 in β‐cells in a diabetic state may greatly help maintain β‐cell function and thus ameliorate symptoms. This highlights the potential therapeutic role of Tipe1 in the management of diabetes symptoms.

Our results showed that Tipe1 enhanced Gαs protein expression and had no effect on its transcription (Figure 4A–C), indicating that Tipe1 might regulate posttranslational modification of Gαs protein. Evidence shows that Tipe1 restrains protein polyubiquitination.^[^ ^50^, ^51^ ^]^ Importantly, the ubiquitin‐proteasome system impaired in islet β cells is directly associated with β cell dysfunction and the incidence of T2D in humans.^[^ ^56^, ^57^, ^58^ ^]^ For the first time, we identified the ubiquitination modification of Gαs protein, and Tipe1 inhibited the K48‐linked polyubiquitination level of Gαs through the deubiquitinase USP5. Interestingly, USP5 directly interacted with target proteins and decreased their K48‐linked polyubiquitination levels.^[^ ^59^, ^60^ ^]^ Our results showed that Tipe1 overexpression significantly promotes the interaction between USP5 and Gαs in a dose‐dependent manner (Figure 5J,K). Furthermore, the knockdown of USP5 remarkably promoted the half‐life of Gαs in Tipe1 overexpressed cells. Although we came to map the critical segment responsible for the interaction of Tipe1 and Gαs, no specific domain was detected. Therefore, it is necessary to further identify the key amino acids of Tipe1 required for interaction with Gαs, and mutations of Tipe1 binding sites in rescue experiments should be performed to support USP5/Gαs as critical downstream signaling events. In addition, it is not clear whether USP5 binds to Gαs depending on Tipe1, and this requires future investigation.

The GLP‐1R and glucose‐dependent insulin polypeptide receptors belong to the GPCR family and act as receptors that stimulate insulin secretion; they have become targets for the treatment of T2D and obesity.^[^ ^61^ ^]^ GLP‐1 mimics promote GLP‐1R activation, stimulate insulin secretion, and increase β cell mass.^[^ ^29^, ^62^ ^]^ It is well known that GLP‐1R agonist (GLP‐1RA) enhances insulin secretion by increasing cAMP production in cells, further demonstrating the important role of Gαs in this regard.^[^ ^30^, ^49^ ^]^ GLP‐1 analogs can only maintain β cell function for a short time under physiological and pathological conditions due to the lack of Gαs expression, resulting in the absence of downstream signals for GLP‐1R.^[^ ^63^, ^64^ ^]^ Our study demonstrated that Tipe1 can increase Gαs protein levels and cAMP signaling by inhibiting ubiquitination degradation of Gαs (Figure 3, 4, 5). Importantly, a decrease in Gαs in β cells has been found to affect GLP‐1R‐dependent regulation of insulin secretion in islets (Figure S9H, Supporting Information), which may have a potential role in improving the therapeutic responsiveness of GLP‐1RA in T2D. The efficacy of GLP‐1RA may be evaluated according to the expression level of Tipe1, and the simultaneous use of both would greatly improve the response of patients with T2D to GLP‐1RA therapy.

However, this study has some limitations. First, we did not perform rescue experiments in *Ins2‐Tipe1BKO* mice because of technical limitations. Second, owing to the limited human samples, studies on human T2D have focused on pancreatic paracancerous tissues from pancreatic cancer patients with diabetes. Therefore, it is important to further determine the expression of Tipe1 in the islets of patients with T2D. Finally, we attempted to rescue the diabetes‐like phenotype of *Ins2‐Tipe1BKO* mice by overexpressing Gαs using an adenovirus in vitro, and the results showed that the insulin secretion level stimulated by high‐glucose concentrations was partially rescued. However, treatment with cAMP agonist could completely reverse the insulin secretion level of *Ins2‐Tipe1BKO* mice, suggesting that there may be unknown mechanisms by which Tipe1 to regulates cAMP, warranting further investigation.

In summary, this study identifies the critical role of Tipe1 in the regulation of β cell homeostasis. For the first time, we report that Tipe1 modulates the ubiquitination modification of Gαs and stabilizes Gαs by inhibiting its polyubiquitination through the deubiquitinase USP5, which improves the downstream level of cAMP. Our study provides a novel candidate target to improve the therapeutic responsiveness of GLP‐1RA in metabolic diseases.

## 4 Experimental Section

### Human Pancreatic Cancer Specimens

Pancreatic cancer specimens from patients with and without diabetes were obtained from the hospital's pathology department. According to the diagnostic criteria for diabetes, there were six patients without diabetes and seven patients with diabetes, without age or sex preference. Information received from all participants before sample collection was kept confidential. Human pancreatic cancer tissue microarrays for immunofluorescence staining were purchased from Shanghai Core Biotechnology Co., Ltd. The use of human pancreatic cancer specimens was approved by the Ethics Committee of the Shandong University School of Basic Medical Sciences (Approval Number: ECSBMSSDU2019‐1‐014), and all participants were informed and provided written informed consent. The relevant data for all human participants are summarized in Table S1 (Supporting Information).

### Mouse Models

Wild‐type (WT) C57BL/6 male mice were provided by the Shandong University Experimental Animal Center (Jinan, China). *Ins2‐Cre* (*RIP‐Cre*) mice (Stock No. 0 03573) were purchased initially from the Jackson Laboratory. *Pdx1‐Cre* mice (Stock No. 01 4647) were purchased from Cyagen Biosciences Inc.*Tipe1‐Loxp* (*Tipe1^f/f^*) mice were generated by Biocytogen Corporation (Beijing, China), as described previously.^[^ ^15^ ^]^ To generate β cell‐specific Tipe1 deficiency mice, Tipe1^f/f^ mice with *Ins2‐Cre* or *Pdx1‐Cre* mice to obtain mice conditioned knockout of Tipe1 in islet β cells (*Ins2^Cre+/−^Tipe1^f/f^* or *Ins2‐Tipe1BKO*; *Pdx1^Cre+/−^Tipe1^f/f^* or *Pdx1‐Tipe1BKO*) were bred. For the diabetes mouse model, after normal diet adaptation, 6‐week‐old mice were fed a high‐fat diet (HFD; 60% Kcal fat) for 4–6 months to induce diabetes. The *db/m* mice were gifted by Prof. Yaoqin Gong, Shandong University, China. *Ins2‐Tipe1BKO* mice with a C57BL/6J background were intercrossed with *db/m* mice to generate double mutations of Tipe1 and Lepr (*Ins2‐Tipe1BKO‐db/db*) or control littermates (*Ins2‐Cre‐db/db*). All animal procedures were performed in accordance with the Guidelines for the Care and Use of Laboratory Animals of Shandong University, and the experiments were approved by the Animal Ethics Committee of Shandong University (Accreditation number: ECSBMSSDU2019‐2‐028).

### Cell Lines and Cell Culture

The MIN6 cell line was obtained by Prof. Ling Gao, ShengLi Hospital, Shandong University (China). It was cultured in Dulbecco's modified eagle medium (DMEM) (Gibco, Thermo Fisher Scientific, Montana, USA) supplemented with 25 mmol L^−1^ glucose, 15% fetal bovine serum (FBS) (Gibco), 2.5 mmol l^−1^ L‐glutamine, 50 mmol l^−1^ β‐mercaptoethanol, 100 U mL^−1^ penicillin, and 100 mg mL^−1^ streptomycin. Human embryonic kidney 293T (HEK293T), Human embryonic kidney 293 (HEK293), and HeLa cell lines were purchased from the Cell Bank of the Chinese Academy of Sciences and cultured in DMEM (Gibco) with 10% FBS (Gibco).

### Viruses

The lentiviral expression vector, Pultra‐Tipe1/Tet‐On‐Tipe1, encoding murine Tipe1 was generated by cloning the corresponding cDNA sequences into the Pultra/Tet‐On vector. The lentiviral expression vector, pLVX‐USP5, was gifted by Prof. Chengjiang Gao, Shandong University, China.

Adenovirus‐overexpressing mouse Gnas (Ad‐Gnas) under the control of the CMV promoter was designed and supplied by WZ Biosciences Inc., China. Adenovirus‐overexpressing mouse Tipe1 (Ad‐Tipe1) under the control of the CMV promoter was designed and supplied by WZ Biosciences Inc., China.

### Isolation of Islet Cells and Culture

Pancreatic islets were isolated and digested with 1 mg mL^−1^ Type V collagenase (Solarbio‐C8170, Beijing, China). After digestion at 37 °C for 30 min, serum was added to neutralize and vigorously shaken for 15 s to disperse the tissues. Hank's buffered saline was used to select the islets under a microscope. After purification at least twice, the islets were collected and transferred to RPMI 1640 medium supplemented with 10% FBS. The islets were cultured overnight in a cell incubator containing 5% CO_2_ and 95% air at 37 °C.

### Fasting‐Refeeding Model

C57BL/6J male mice were fed a normal diet until fasting and re‐feeding treatments were started. The mice were randomly divided into three groups: i) the normal group, where mice were untreated; ii) the fasting group, where mice were put on fasting for 24 h; and iii) the refeeding group, where mice were put on fasting for 24 h and then fed a normal diet for 12 h.

### Transfection and Infection

For gene silencing in MIN6, HEK293T, or HEK293 cells, the cells were transfected with the respective 50 nM siRNA mixtures using Lipofectamine 2000 reagent (Invitrogen, USA) for 72 h.

MIN6 cells were first cultured to 70% confluence in a complete medium and then cultured in a fresh medium containing the lentivirus for 16–24 h. After infection, a culture medium containing 10 µg mL^−1^ puromycin was used for screening.

For adenovirus infection, MIN6 cells were cultured in a complete medium and transferred to 12‐well plates at a density of 1 × 10^6^ cells well^−1^; next, 1 × 10^10^ pfu of the virus was added. After 12 h, the medium was replaced with a fresh complete medium, and the cells were cultured for another 24–48 h. Islets were cultured in a complete medium at a density of ≈30 islets/well in a 96‐well plate, and 1 × 10^8^ pfu of the virus was added. After 12 h, the medium was replaced with a fresh complete medium and the cells were cultured for another 24–48 h (**Table** **1**).

**Table 1:** Sequences of siRNA.

Names	Sequences(5′→ 3′)
Murine *Tipe1* siRNA	CUCUUGUUGUACCAGACUA
Murine *GNAS* siRNA	CUGCAUGUUAAUGGGUUUATT
Murine *GPRASP1* siRNA	GGCCAAACAAGAGGCAAAUTTT
Murine *USP5* siRNA	GGAAUUCUUCCUACAUCUU
Human *Tipe1* siRNA	GCAUCUGUAGCAGCUGUUU
Human *USP5* siRNA	GGAGUUCUUCCUUCACCUU
Human *USP9X* siRNA	GGACUUCUUUGAAAGUAAUTT
Human *MAEA* siRNA	CCGCUCAGAAGAACAUUGATT
Human *WWP2* siRNA	AGGAGGTTCTGCCTGTAATT

### Glucose Tolerance Test (GTT), Glucose‐Stimulated Insulin Secretion (GSIS) and Insulin Tolerance Test (ITT)

The GTT was performed by intraperitoneal injection of D‐glucose (2 g kg^−1^ body weight) under fasting conditions for 16 h. Blood glucose levels were determined at the indicated time points after glucose administration by using a glucometer (Yicheng, China).

For GSIS, the mice were treated as GTT. After glucose injection, mouse orbital blood was collected at a specified time point, and blood insulin levels were measured using a mouse insulin enzyme‐linked immunosorbent assay (ELISA) kit (Elabscience, China). For in vitro GSIS, mouse islets (30 per well in 48 well plates) were placed in 2.5 mM glucose medium overnight. They were then washed and incubated in KRBH buffer (containing 0.2% BSA supplemented with 2.5 mM glucose and 10% FBS) at 37 °C for 1 h. The islets were then cultured with either 2.8 mM or 16.7 mM glucose for 1 h. Insulin levels were determined using an ELISA kit (CCM, USA). Insulin secretion was standardized based on total cellular protein content.

For ITT, mice were put on fasting for 4–6 h followed by intraperitoneal injection of human insulin (Sigma‐Aldrich) at 0.7 U kg^−1^ body of weight. Blood glucose levels were monitored at specific times after insulin injection.

### Immunohistochemical (IHC) and Immunofluorescence (IF) Staining

Pancreatic tissues were fixed with 4% paraformaldehyde and sliced (3–5 µm) after paraffin embedding. IHC was performed using the EnVision+System‐horse radish peroxidase (HRP) (DAB) kit (Dako, USA) for target antibody or control IgG staining. For IF staining, the primary antibody was treated at 4 °C overnight, and the secondary antibody was treated with Alexa Fluor 594 Goat; Anti‐guinea Pig IgG (#ab150188, Abcam, Cambridge, UK); Alexa Fluor 594 Goat Anti‐Mouse IgG (#SA00006‐3, Proteintech, Illinois, USA) and Alexa Fluor 488 Goat Anti‐Rabbit IgG (#SA00006‐2, Proteintech, Illinois, USA) at 37 °C for 1 h. Finally, the nuclei were stained with DAPI. Microscopic analyses were performed using an Olympus IX51 microscope (Tokyo, Japan) and ImageJ software.

### Multiplexed Immunofluorescence Staining and Image Analysis

Multiplexed immunofluorescence staining of pancreatic tissue slides was performed using Opal Chemistry (PerkinElmer, USA). The antibodies used were grouped into two panels. Panel 1 contained antibodies for TIPE1 (#BS60529, Bioworld technology), insulin (#sc‐377071, Santa Cruz Biotechnology, Dallas, Texas, USA), glucagon (#sc‐514592, Santa Cruz Biotechnology), and somatostatin (#sc‐74556, Santa Cruz Biotechnology); Panel 2 contained antibodies for TIPE1(Bioworld technology), insulin (Santa Cruz Biotechnology), Gαs (#sc‐55545, Santa Cruz Biotechnology), and USP5(#sc‐390943, Santa Cruz Biotechnology). After deparaffinization, the slides were processed in antigen retrieval buffer with microwave irradiation (4 min at 100% power, 15–20 min at 20% power) and blocked with 40% goat serum for 10–30 min at room temperature. Slides were incubated in the primary antibody at 37 °C for 30–60 min, then washed with Tris‐buffered saline (TBST); they were then incubated with the HRP‐conjugated secondary antibody at room temperature for 10 min, then washed with TBST buffer solution. Thereafter, the slides were incubated with Opal working buffer for 10–20 min at room temperature and washed with TBST. The above steps were repeated for other antibodies. The antibodies were removed by microwave treatment and a new round of staining was performed. Finally, all the nuclei were stained with DAPI.

We used TissueFAXS spectral system and StrataQuest analysis software (TissueGnostics, Vienna, Austria) to analyze the multichannel immunofluorescence image and perform statistical analysis. In this study, all islets in human pancreatic cancer specimens were counted.

For multiplexed immunofluorescence staining, MIN6 cells grown on coverslips were washed with PBS, fixed with 4% paraformaldehyde, and permeabilized with 0.2% Triton X‐100 for 10 min. After blocking with 5% BSA for 30 min, MIN6 cells were stained with antibodies and then incubated with the HRP‐conjugated secondary antibody. Thereafter, the cells were incubated with Opal working buffer for 10 min at room temperature. The antibodies were removed by antibody eluent (#abs994, Absin) at 37 °C for 25 min and a new round of staining was performed. Finally, all the nuclei were stained with DAPI. Confocal microscopy was performed using a Zeiss LSM 980 laser confocal microscope. The co‐localization was analyzed by ImageJ software.

### cAMP Measurement

To measure cAMP levels in MIN6 cells, phosphate‐buffered saline (PBS) was added to MIN6 cells and repeatedly freeze‐thawed, centrifuged at 10 000 g for 10 min, and the particles were removed. MIN6 cells were repeatedly freeze‐thawed in PBS and centrifuged 10 000 g for 10 min to remove particles. The supernatants were collected and the cAMP content was determined using cAMP ELISA Kit (Elabscience, China) according to the manufacturer's instructions.

### β‐Cell Area and Mass Measurement

To measure β‐cell mass, the whole pancreas was removed, weighed, and fixed in 4% paraformaldehyde overnight at 4 °C. Next, it was transferred to 70% ethanol at 4 °C. Next, the fixed pancreases were processed into 3‐µm thick paraffin sections. The sections were dewaxed and hydrated, and then, antigen retrieval was performed. The pancreatic sections were stained with an anti‐insulin antibody for IHC or IF. Pancreatic sections were imaged using a microscope (Olympus). ImageJ software was used to measure β‐cell according to insulin‐positive areas and pancreatic areas. Islet β cell mass was measured using the following formula:

(1) $$\frac{total\;insulin\;positive\;area}{total\;pancreatic\;area}\times pancreatic\;tissue\;weight$$

### Western Blotting

RIPA buffer (Biotechnology) was used to lyse cells and tissues. Equal amounts (20–30 µg) of proteins were subjected to sodium dodecyl‐sulfate polyacrylamide gel electrophoresis (SDS‒PAGE) and transferred onto a polyvinylidene difluoride (PVDF) membrane filter. Slides were incubated with the appropriate antibodies against TIPE1 (#BS60529, Bioworld technology), PCNA (#ab92552, Abcam), MAFA (#sc‐390491, Santa Cruz Biotechnology), PDX1 (#sc‐390792, Santa Cruz Biotechnology), GLUT2 (#sc‐518022, Santa Cruz Biotechnology), Gαs (#sc‐55545, Santa Cruz Biotechnology), USP5 (#sc‐390943, Santa Cruz Biotechnology), HA (#M180‐3, MBL), DDDK (#M185‐3, MBL), Myc (#18 583, CST), GFP (#66002‐1‐Ig, Proteintech), GASP1 (#A16090, ABclonal), PKA (#AF7746, Affinity), p‐PKA (Thr198) (#AF7246, Affinity), CREB (#9197S, CST), p‐CREB (Ser133) (#9198, CST), AMPK (#ab80039, Abcam), P‐AMPK (Thr172) (#2535S, CST), AKT (#ab18785, abcam), p‐AKT (S473) (#4058s, CST), p‐AKT (T308) (#13038S, CST), IR (#sc57342, Santa Cruz Biotechnology), p‐IR (Tyr1150/1151) (#sc‐81500, Santa Cruz Biotechnology), Caspase 3 (#14 220, CST), Caspase 9 (#9508S, CST), Bcl‐2 (#ab182858, Abcam), Ub (#3936, CST), Ub‐K48 (#4289S, CST), Ub‐K63 (#5621S, CST), MAEA (#abs133634, Absin Biotechnology), WWP2 (#sc‐398090, Santa Cruz Biotechnology), USP9X (#sc‐365353, Santa Cruz Biotechnology), β‐actin (#66009‐1‐Ig, Proteintech), and GAPDH (#60004‐1‐Ig, Proteintech). Next, they were incubated with secondary antibodies, Rabbit Anti‐Goat IgG H&L (HRP) (#SA00001‐2, Proteintech) and Mouse Anti‐Goat IgG H&L (HRP) (#SA00001‐1, Proteintech).

### Coimmunoprecipitation (Co‐IP)

The cell lysates were incubated with anti‐HA, anti‐Flag, anti‐Tipe1, anti‐USP5 or anti‐Gαs antibodies, and rotated overnight at 4 °C. The lysates were then, incubated with protein A/G magnetic beads for 4 h. The samples were then washed five with PBST buffer. The 1 × SDS sample buffer was added to the magnetic beads, and the protein was denatured at 100 °C for 5 min. SDS‐PAGE Western blotting analysis was performed.

### Quantitative RT‐PCR (qRT‐PCR)

Total RNAs were extracted from the pancreatic islets or other organs of mice and from MIN6 β cells using TRIzol reagent (Invitrogen). cDNA was synthesized using the RevertAid First Strand cDNA Synthesis Kit (Thermo Fisher Scientific). Real‐time quantitative PCR (qPCR) was performed using the SYBR Green PCR reagent (TIANGE). The relative mRNA level of the target gene was calculated using the 2^(−△△^ *^Ct^* ^)^ method. The △*Ct* was computed with normalization to β‐actin, which served as the internal control (**Table** **2**).

**Table 2:** Sequences of real‐time PCR primers.

Species	Name	Forward (5′→3′)	Reverse (5′→3′)
Mouse	*Tipe1*	CAGAACCCATGGACACCTTC	CTTCGTGGCCTGGTACAGTT
Mouse	*Ins1*	CGTGGCTTCTTCTACACACCCA	TGCAGCACTGATCCACAATGCC
Mouse	*Ins2*	CTGCTGGCCCTGCTCTTC	AACCACAAAGGTGCTGCTTGA
Mouse	*Pdx1*	TTCCCGAATGGAACCGAGCCTG	TTTTCCTCGGGTTCCGCTGTGT
Mouse	*Mafa*	TTCAGCAAGGAGGAGGTCAT	CCGCCAACTTCTCGTATTTC
Mouse	*Gcg*	CCTTCAAGACACAGAGGAGAACC	CTGTAGTCGCTGGTGAATGTGC
Mouse	*Pax6*	CTGAGGAACCAGAGAAGACAGG	CATGGAACCTGATGTGAAGGAGG
Mouse	*Slc2A2*	GTTGGAAGAGGAAGTCAGGGCA	ATCACGGAGACCTTCTGCTCAG
Mouse	*Pcna*	CAAGTGGAGAGCTTGGCAATGG	GCAAACGTTAGGTGAACAGGCTC
Mouse	*Ccnd1*	GCAGAAGGAGATTGTGCCATCC	AGGAAGCGGTCCAGGTAGTTCA
Mouse	*Ki67*	GAGGAGAAACGCCAACCAAGAG	TTTGTCCTCGGTGGCGTTATCC
Mouse	*Gnas*	ACCAGCGCAACGAGGAGAA	CCCATTAACATGCAGGATCCTC
Mouse	*Gprasp1*	GGAGTCCACTATGGCAGATAGG	TGACAGGTCTCAGAGTCCACAC

### Protein Half‐Life Assay

Transfected HEK293T or HEK293 cells were treated with 500 µg mL^−1^ cycloheximide (Selleck) for a specified time. Cell lysate was collected and Gαs protein levels were analyzed by Western blotting. Gαs protein expression was normalized to GAPDH at different time points using ImageJ software.

### Flow Cytometry

MIN6 cells were pretreated, washed thrice with PBS, collected, and stained using an EdU assay kit (Beyotime Biotechnology) or anti‐Ki67‐PE (eBioscience) antibody. Data were obtained using CytoFLEXS flow cytometer (BD Biosciences) and analyzed using CytExpert.

### Statistical Analysis

All statistical analyses were performed using GraphPad Prism 8.0 (GraphPad Software, San Diego, CA, USA). Two‐tailed unpaired Student's *t*‐tests between two groups were used to determine significance. All data in the figures were presented as the mean ± SEM (standard error of the mean). Statistical significance was reported as ^*^ *p* < 0.05; ^**^ *p* < 0.01; ^***^ *p* < 0.001; and n.s., no significance.

## Conflict of Interest

The authors declare no conflict of interest.

## Author Contributions

L.D. performed the research, interpreted the data, and wrote the manuscript. Y.S. provided experimental skills and assisted in data analysis. Y.L. provided experimental skills in vivo experiments. J.Z., Z.F, C.R., and W.L. were involved in the construction of the plasmid and experimental skills in vitro experiments. J.Z., R.X., Y.F. contributed to multiplexed immunofluorescence staining and image analysis. H.W. helped with experiments in mice. Z.Z. provided data analysis of ubiquitin experiments. C.M., X.L., X.Y., Z.W., C.L., and P.L. provided careful guidance and assistance for this project. L.G. was in charge of the study design, data analysis, work organization and supervision, and manuscript review. All authors discussed the results and commented on the manuscript.

## Supporting information

Supporting Information

## Data Availability

Research data are not shared.
