# Supplementary material for: Beta‐Cell Tipe1 Orchestrates Insulin Secretion and Cell Proliferation by Promoting Gαs/cAMP Signaling via USP5
Source: Adv Sci (Weinh). 2024 Feb 28;11(16):2304940. doi: 10.1002/advs.202304940 (PMC11040358; doi:10.1002/advs.202304940)
Supplement: Supplementary file 1 — Supporting Information [file ADVS-11-2304940-s001.pdf]

ADVANCED SCIENCE
Open Access

Supporting Information

for *Adv. Sci.*, DOI 10.1002/advs.202304940

Beta-Cell Tipe1 Orchestrates Insulin Secretion and Cell Proliferation by Promoting Gαs/cAMP Signaling via USP5

*Lu Ding, Yang Sun, Yan Liang, Jie Zhang, Zhendong Fu, Caiyue Ren, Pengfei Li, Wen Liu, Rong Xiao, Hao Wang, Zhaoying Zhang, Xuetian Yue, Chunyang Li, Zhuanchang Wu, Yuemin Feng, Xiaohong Liang, Chunhong Ma and Lifen Gao**

Supplementary Materials

Table S1. Blood glucose levels of pancreatic cancer specimens from patients with and without type 2 diabetes (T2D).

Sample	ND/T2D	Gender	FBG (mM)
1	ND	Female	5.61
2	ND	Male	6.08
3	ND	Male	5.57
4	ND	Female	5.18
5	ND	Female	5.37
6	ND	Male	5.99
7	T2D	Male	7.42
8	T2D	Male	13.13
9	T2D	Male	9.34
10	T2D	Female	6.75
11	T2D	Male	17.49
12	T2D	Female	6.91
13	T2D	Male	12.32

Supplementary Figures

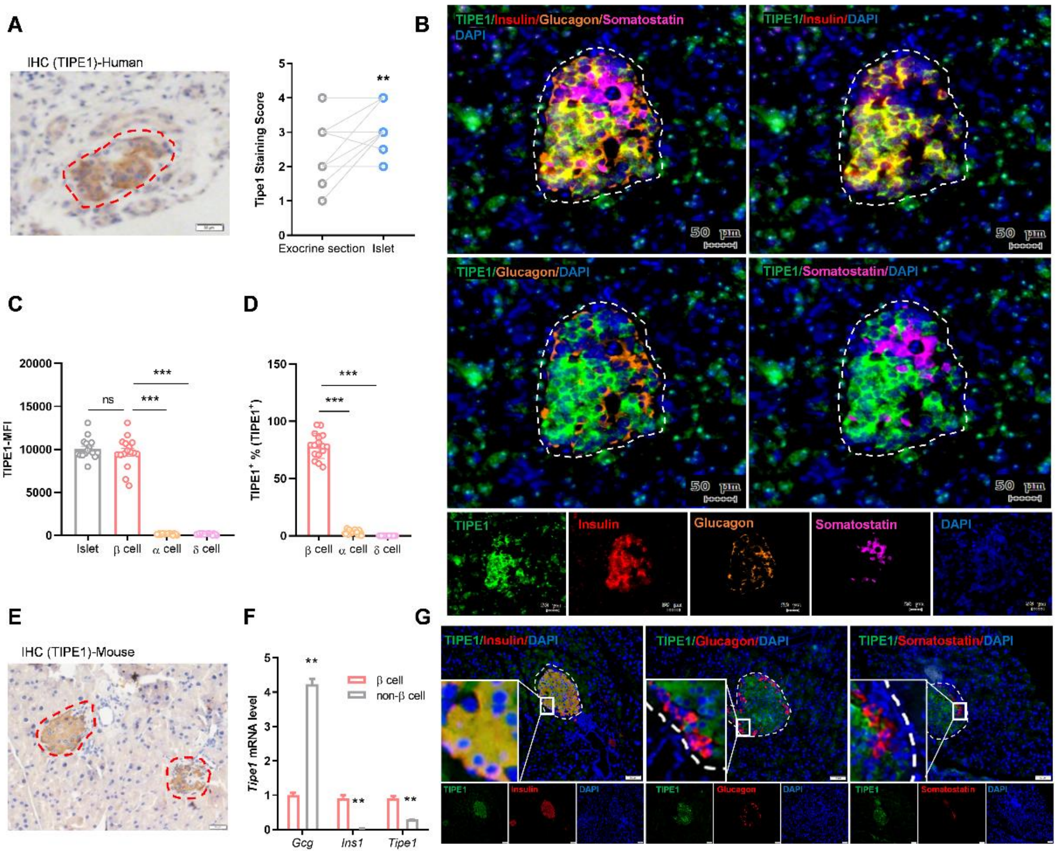

Figure S1. Tipe1 is highly expressed in pancreatic islet β cells. A) Human pancreatic tissue microarray of patients with pancreatic cancer was used to perform IHC staining with anti-TIPE1 antibody and paracancerous tissues were analyzed. Scale bar, 50 μm. (n = 12). B) Representative images for multiplexed immunofluorescence staining (TIPE1, green; Insulin, red; Glucagon, orange; Somatostatin, pink) in paracancerous tissues from patients with pancreatic cancer. C,D) The median fluorescence intensity (MFI) and frequency for the expression of Tipe1 in β, α, and δ cells in human islet tissues of paracancerous tissues from patients with pancreatic cancer. (n = 16). E) Sections of pancreas tissues from 12-week-old WT mice were used to perform IHC staining with anti-TIPE1 antibody. Nuclei were stained with Hematoxylin (blue). Scale bar, 50 μm. F) Total RNAs isolated from β cells and non-β-cells of 8—12-week-old WT mice were subjected to qRT-PCR analysis for the indicated genes. mRNA levels were normalized to *β-actin* mRNA. (n =10). G) IF staining of β cells (insulin, red), α cells (glucagon, red) and δ cells (somatostatin, red) in islets of 3-month-old male WT mice. Nuclei were stained with DAPI (blue) and TIPE1-positive cells were stained in green. Scale bar, 50 μm. Data are presented as the mean ± SEM. Data were statistically analyzed by Student's *t* test. $^{**}P<0.01$, $^{***}P<0.001$, ns indicates no significant difference.

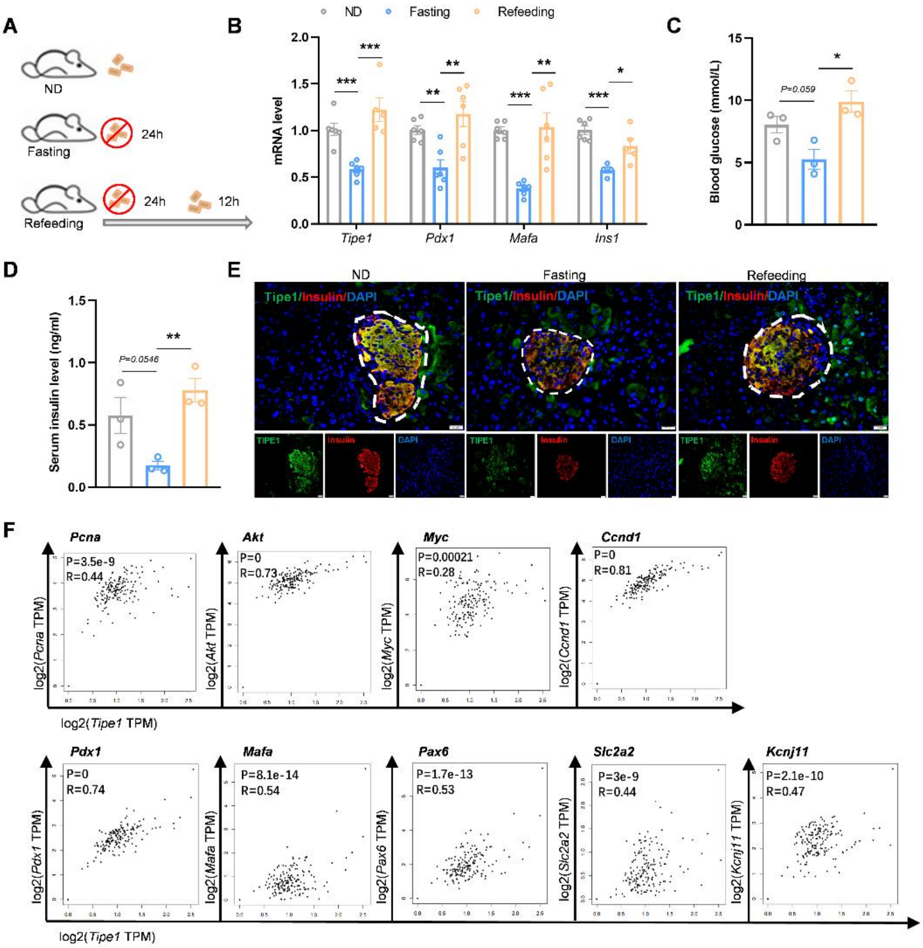

Figure S2. Tipe1 expression in pancreatic islet is reduced in fasting status, and restored after refeeding. A) Schematic description of a fasting-refeeding model. B) Total RNAs isolated from islets were subjected to qRT-PCR analysis for gene expression. Gene mRNA was normalized to *β-actin* mRNA. (n = 6 mice/group). C) Blood glucose levels of mice. (n = 3 mice/group). D) Serum insulin levels of mice. (n = 3 mice/group). E) IF staining of pancreatic sections of fasting and refeeding mice with anti-TIPE1 and anti-Insulin antibodies. Scale bar, 50 µm. F) GEPIA database was used to analyze the relationship between *Tipe1* and proliferation or insulin regulation related genes in pancreas of healthy individuals.

Data are presented as the mean ± SEM. Data were statistically analyzed by Student's *t* test. **P*<0.05, ***P*<0.01, ****P*<0.001, ns indicates no significant difference.

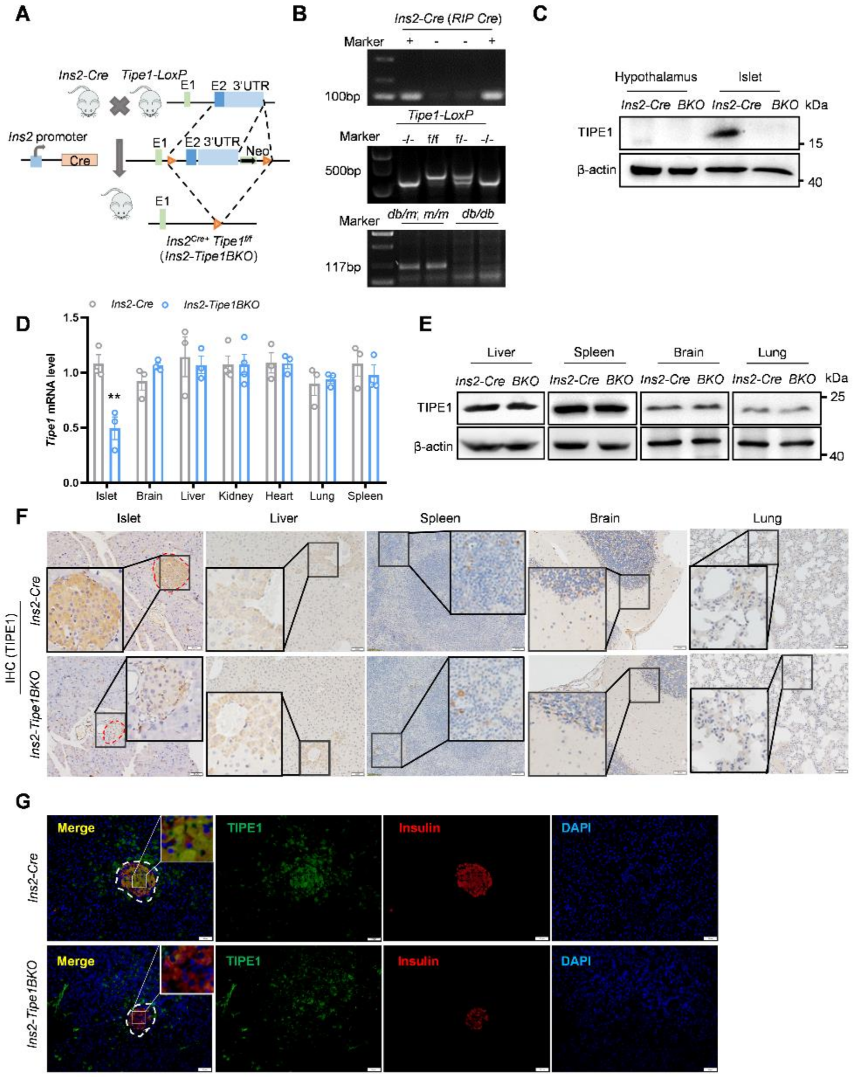

Figure S3. Characterization of *Ins2-Tipe1BKO* mice. A) Strategy of islet β cell specific *Tipe1*-knockout mice. B) Schematic description of genotyping. C) Protein extracts isolated from hypothalamus and islet tissues of 8－12-week-old *Ins2-Cre* and *Ins2-Tipe1BKO* mice were subjected to Western blotting analysis for TIPE1 expression. D) Total RNAs isolated from the pancreas of 8－12-week-old *Ins2-Cre* and *Ins2-Tipe1BKO* mice were subjected to qRT-PCR analysis for Tipe1 expression. *Tipe1* mRNA was normalized to *β-actin* mRNA. (n = 3 mice/group). E) Proteins isolated from different tissues of *Ins2-Cre* and *Ins2-Tipe1BKO* mice were subjected to Western blotting analysis for Tipe1 expression. F) Indicated tissues of 12-week-old mice were subjected to IHC staining for TIPE1 expression. G) IF staining of pancreatic sections of *Ins2-Cre* and *Ins2-Tipe1BKO* mice with anti-TIPE1 and anti-Insulin antibodies. Scale bar, 50 μm.

Data are presented as the mean ± SEM. Data were statistically analyzed by Student's *t* test. ***P*<0.01, ns indicates no significant difference.

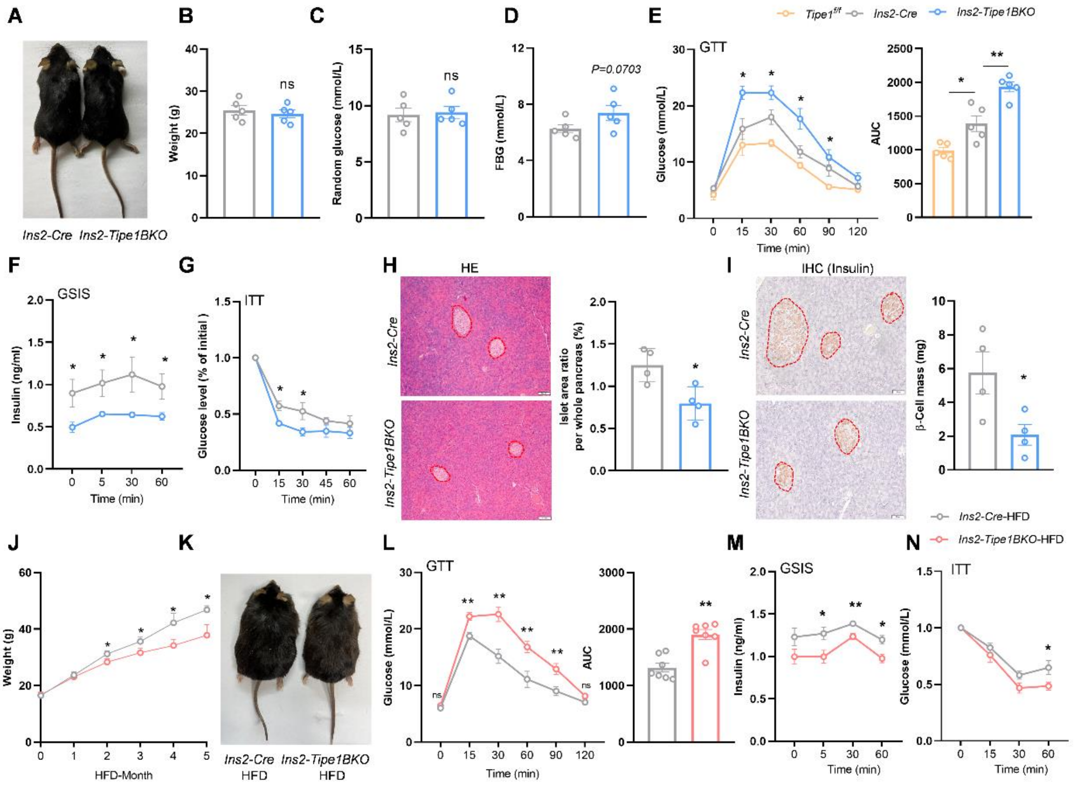

Figure S4. Deletion of Tipe1 in β cells impairs glucose homeostasis.
(A) Overall appearance of *Ins2-Cre* and *Ins2-Tipe1BKO* mice. B) Body weight of *Ins2-Cre* and *Ins2-Tipe1BKO* mice. (n = 5 mice/group). C,D) Random blood glucose and FBG levels were detected in 3-month-old male *Ins2-Cre* and *Ins2-Tipe1BKO* mice. (n = 5 mice/group). E) Three-month-old male *Tipe1$^{f/f}$*, *Ins2-Cre* and *Ins2-Tipe1BKO* mice were fasted for 16 h and intraperitoneally injected with glucose (2 g/kg body weight). Blood glucose levels (left panel) and area under the curve (AUC) (right panel) during the intraperitoneal glucose tolerance test (GTT). (n = 5 mice/group). F) Mice treated as in E. Blood insulin levels were measured by ELISA at 0, 5, 30, and 60 min after glucose injection (GSIS). (n = 6 mice/group). G) Mice fasted for 6 h were intraperitoneally injected with a single dose of recombinant human insulin (0.7 U/kg body weight), and blood glucose levels were detected at the indicated time points (ITT). Results were normalized by initial blood glucose concentration. (n = 5 mice/group). H) H&E staining was performed in pancreatic tissues of *Ins2-Cre* and *Ins2-Tipe1BKO* mice, followed by measurements of the islet area/pancreatic area ratio. (n = 4 mice/group). I) IHC staining was used to detect insulin in pancreatic sections, followed by measurements of β-cell mass. (n = 4 mice/group). J) Growth curves of male *Ins2-Tipe1BKO*-HFD mice and *Ins2-Cre*-HFD mice. (n = 3 mice/group). K) Photograph of 5-month-old *Ins2-Tipe1BKO*-HFD mice and control *Ins2-Cre*-HFD mice. L) Plasma glucose levels during the glucose tolerance test in *Ins2-Cre* and *Ins2-Tipe1*BKO mice fed an HFD for 5 months. (n = 7 mice/group). M) Blood insulin levels during GSIS-test for mice fed an HFD for 5 months. (n = 6 mice/group). N) Plasma glucose levels during the ITT-test in *Ins2-Cre*

and *Ins2-Tipe1*BKO mice fed an HFD for 5 months. Results were normalized by initial blood glucose concentration. (n = 7 mice/group).

Data are presented as the mean ± SEM. Data were statistically analyzed by Student's *t* test. **P*<0.05*,* ***P*<0.01, ns indicates no significant difference.

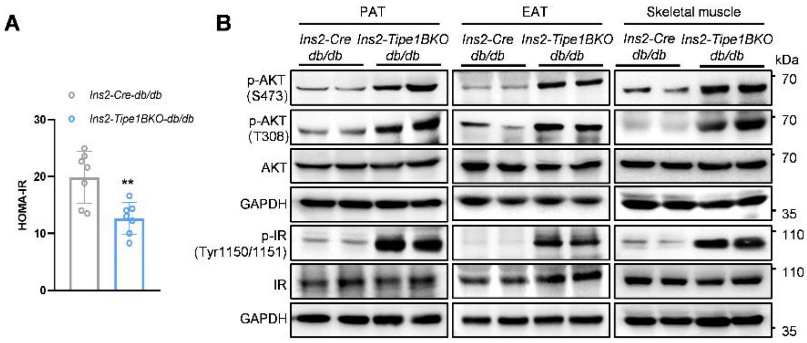

Figure S5. The HOMA-IR and improved insulin signaling in *Ins2-Tipe1BKO-db/db* mice. A) The HOMA-IR of *Ins2-Cre-db/db* and *Ins2-Tipe1BKO-db/db* mice (n = 7 mice/group). B) Proteins isolated from perirenal adipose tissue (PAT), epididymal adipose tissue (EAT) and skeletal muscles of *Ins2-Cre-db/db* and *Ins2-Tipe1BKO-db/db* mice were subjected to Western blotting analysis for p-AKT, AKT, p-IR and IR expression.

Data are presented as the mean ± SEM. Data were statistically analyzed by Student's *t* test. ***P*<0.01, ns indicates no significant difference.

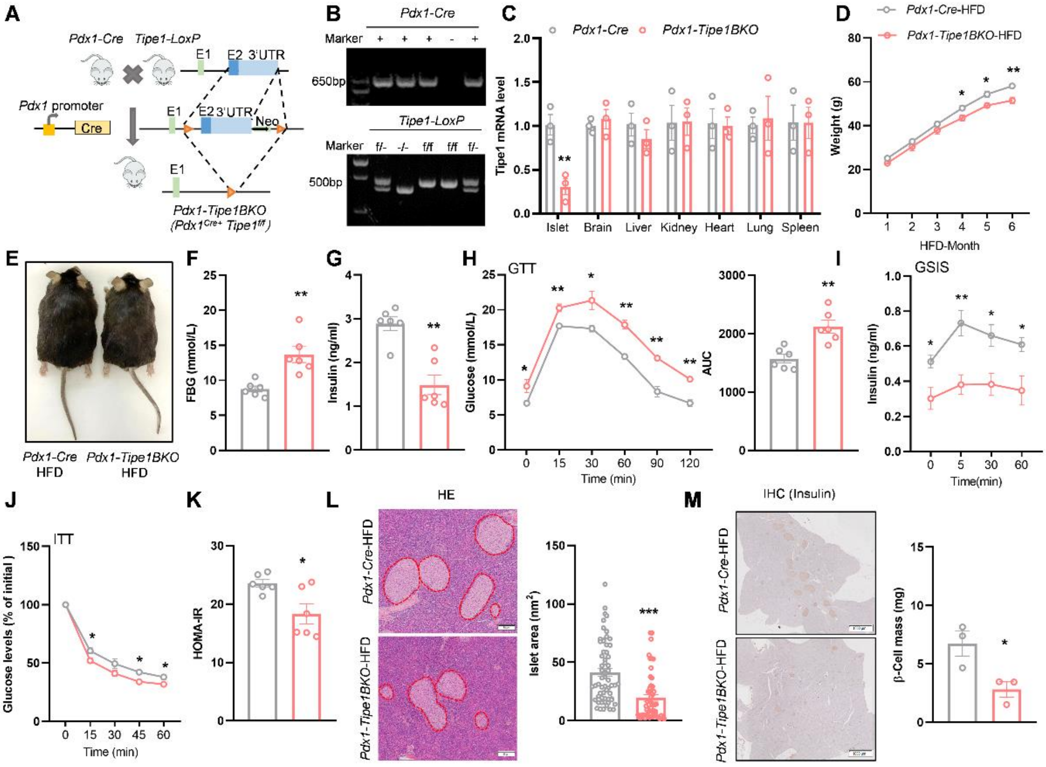

Figure S6. Impaired glucose homeostasis in Tipe1-deficient mice in HFD induced diabetes. *Pdx1-Cre*-HFD and *Pdx1-Tipe1BKO*-HFD mice were fed a HFD for 6 months. A) Strategy of islet β cell-specific *Tipe1*-knockout mice. B) Schematic description of genotyping. C) Total RNAs isolated from the indicated tissues of 8~12-week-old *Pdx1-Cre* and *Pdx1-Tipe1BKO* mice were subjected to qRT-PCR analysis for Tipe1 expression. *Tipe1* mRNA was normalized to *β-actin* mRNA. (n = 4 mice/group). D) Growth curves of male *Pdx1-Cre* -HFD and *Pdx1-Tipe1BKO*-HFD mice. (n = 6 mice/group). E) Photograph of *Pdx1-Cre*-HFD and *Pdx1-Tipe1BKO*-HFD mice. F,G) FBG and fasting insulin level. (n = 6 mice/group). H) Plasma glucose levels during the GTT-test. (n = 6 mice/group). I) Blood insulin levels during GSIS-test. (n = 6 mice/group). J) Plasma glucose levels during the ITT-test. (n = 6 mice/group). K) HOMA-IR. (n = 6 mice/group). L) H&E staining was performed in pancreatic tissues, followed by measurements of the islet area. (n = 3 mice/group). M) IHC staining of insulin. (n = 3 mice/group).

Data are presented as the mean ± SEM. Data were statistically analyzed by Student's *t* test. **P*<0.05, ***P*<0.01, ****P*<0.001, ns indicates no significant difference.

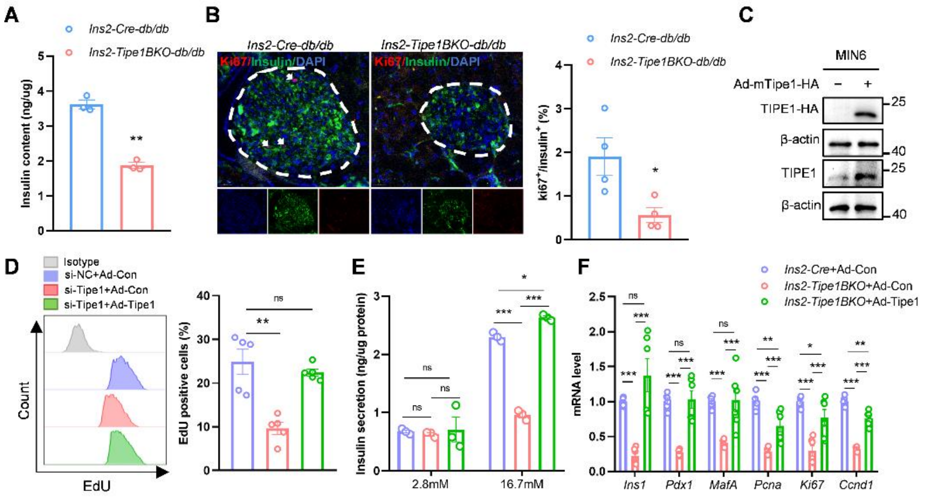

Figure S7. Tipe1 deficiency reduces β cell intracellular insulin and proliferation. A) Pancreatic islets from 16-week-old *Ins2-Cre-db/db* and *Ins2-Tipe1BKO-db/db* mice were detected intracellular insulin level. (n = 3 mice/group). B) IF staining of Ki67. Ki67-positive cells in islets were normalized to total insulin-positive cells in the same area. Scale bar, 50 µm. (n = 4 mice/group). C) MIN6 cells were infected with Ad-Tipe1 adenovirus for 48 h, and the protein level of TIPE1 and TIPE1-HA were detected by Western blotting. D) MIN6 cells were silenced Tipe1 for 24 h, then infected with either Ad-Con or Ad-Tipe1 for 48 h, were subjected to EdU incorporation assays by flow cytometry. Flow cytometry histograms showing the level of EdU in MIN6 cell line. (n = 5). E) Pancreatic islets from 8-week-old *Ins2-Cre* and *Ins-Tipe1BKO* mice were treated with either Ad-Con or Ad-Tipe1 for 48 h, subsequently treated with 2.8 or 16.7 mM glucose in an in vitro setting. Insulin levels in the culture supernatants were determined by ELISA. (n = 3 mice/group). F) Islets were treated as in E, and qRT-PCR was performed to detect the indicated gene expression. mRNA levels were normalized to *β-actin* mRNA (n = 5 mice/group). Data are presented as the mean ± SEM. Data were statistically analyzed by Student's *t* test. **P*<0.05*,* ***P*<0.01. ****P*<0.001, ns indicates no significant difference.

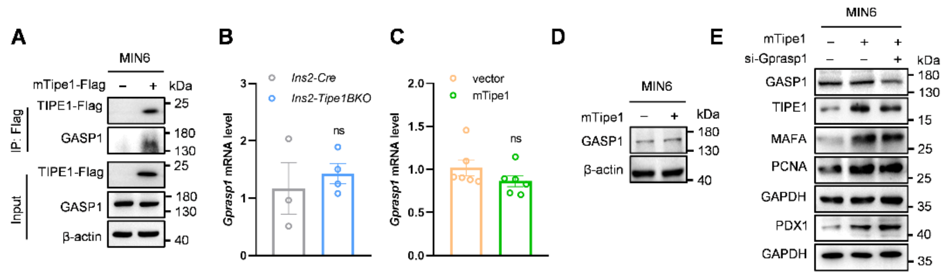

Figure S8. Tipe1 regulates β cell function not through Gprasp1. A) Intracellular interaction between Tipe1 and GASP1, followed by IP with anti-Flag, probed with anti-GASP1. B) *Gprasp1* expression in islets of *Ins2-Cre* and *Tipe1BKO* mice was measured by qRT-PCR. mRNA levels were normalized to *β-actin* mRNA. (n/ *Ins2-Cre* = 3; n/ *Ins2-Tipe1BKO* = 4). C,D) *Gprasp1* expression of MIN6 cells infected with either Pultra-NC or Pultra-Tipe1 lentiviruses was measured by qRT-PCR and Western blotting. mRNA levels were normalized to *β-actin* mRNA. (n = 6). E) MIN6 cells were infected with either Pultra-NC or Pultra-Tipe1 lentiviruses and silenced *Gprasp1* for 48 h, protein levels were detected by Western blotting.

Data are presented as the mean ± SEM. Data were statistically analyzed by Student's *t* test. ns indicates no significant difference.

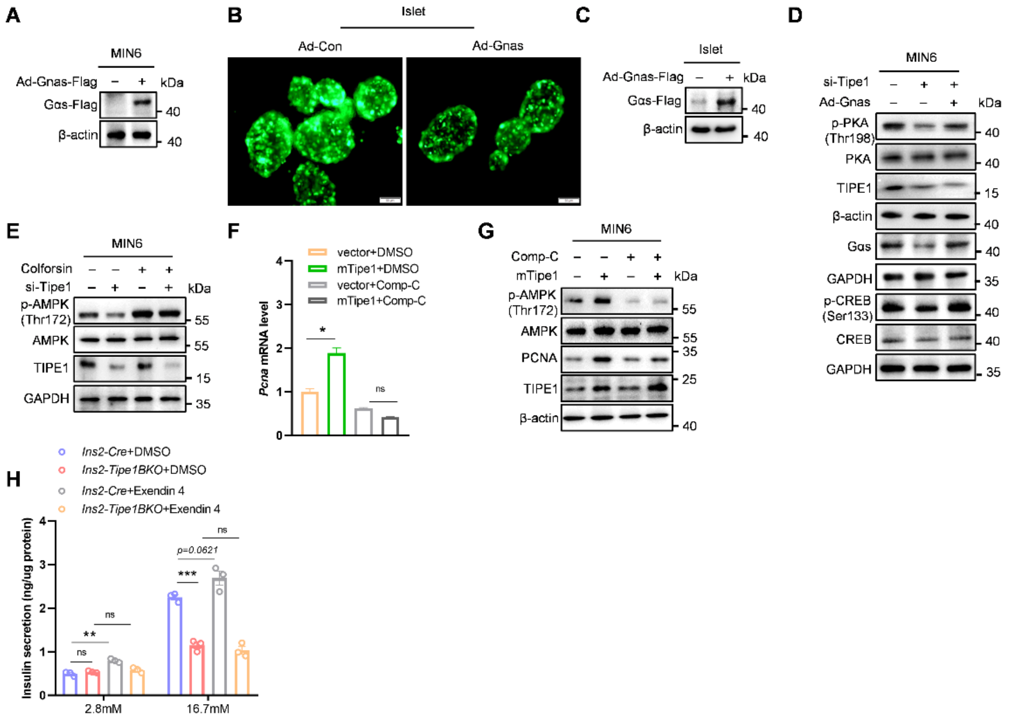

Figure S9. Tipe1 activates the cAMP/PKA/CREB pathway in MIN6 cells. A) MIN6 cells were infected with Ad-Gnas adenovirus for 48 h, and the protein level of Gαs was detected by Western blotting. B) The fluorescence was observed in islets infected with Ad-Gnas adenovirus. C) The protein level of Gαs in islets infected with Ad-Gnas adenovirus for 48 h was detected by Western blotting. D) MIN6 cells were silenced *Tipe1* 24 h after infected with Ad-Gnas adenovirus for 48 h, and the protein levels of PKA, p-PKA (Thr198), CREB and p-CREB (Ser133) were detected by Western blotting. E) MIN6 cells silenced *Tipe1* for 48 h, then treated with cAMP agonist (Colforsin, 10 μM) for 48 h. The protein levels of p-AMPK and AMPK were detected by Western blotting. F,G) MIN6 cells silenced *Tipe1* for 48 h, then treated with p-AMPK inhibitor (Comp-C, 20μM) for 48 h. PCNA expression was detected by qRT-PCR and Western blotting. mRNA levels were normalized to *β-actin* mRNA. H) Pancreatic islets from 8-week-old *Ins2-Cre* and *Ins2-Tipe1BKO* mice were treated with GLP-1 receptor agonist (exendin 4, 100 nM) for 48 h, subsequently treated with 2.8 or 16.7 mM glucose in an in vitro setting. Insulin levels in the culture supernatants were determined by ELISA. (n = 3 mice/group).

Data are presented as the mean ± SEM. Data were statistically analyzed by Student's *t* test. **P*<0.05*,* ***P*<0.01, ****P*<0.001, ns indicates no significant difference.

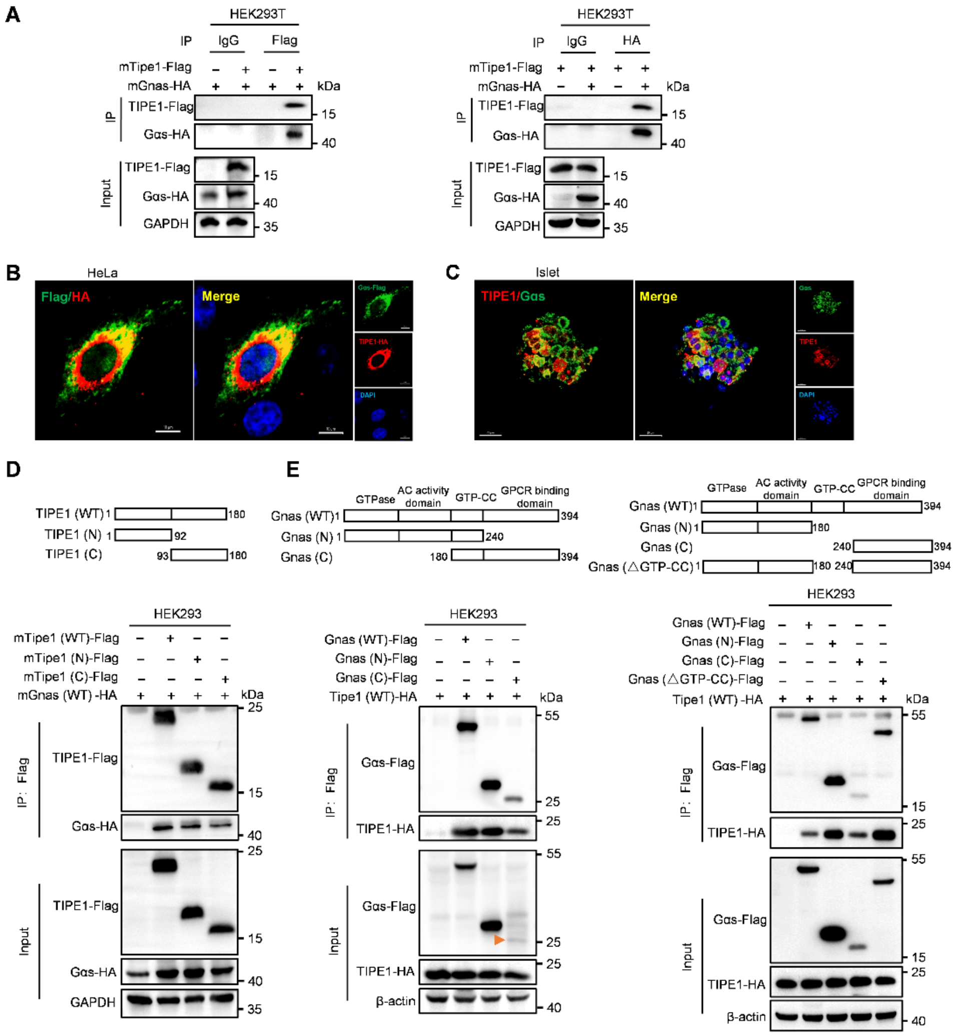

Figure S10. Both N and C-terminal of Tipe1 interacts with Gαs. A) HEK293T cells expressing mTipe1-Flag and mGnas-HA. Gnas-HA was immunoprecipitated from HEK293T cell lysates and immunoblotted with anti-Flag and anti-HA, respectively. B) HeLa cells were transfected with human Tipe1-HA and Gnas-Flag overexpression plasmid. Then, the cells were subjected to IF staining with antibodies against HA (red), Flag (green). Nuclei were stained with DAPI (blue). Confocal assay was performed. Scale bar, 10 μm. C) Islets from 12-week-old WT mice were subjected to IF staining with antibodies against TIPE1 (red), Gαs (green). Nuclei were stained with DAPI (blue). Confocal assay was performed. Scale bar, 20 μm. D,E) Flag-Tagged TIPE1, Gnas and their truncates were constructed and co-transfected into HEK293 cells, and cell lysates were precipitated with anti-Flag antibody and immunoblotted with anti-HA antibody as indicated (The orange arrow indicates the band).

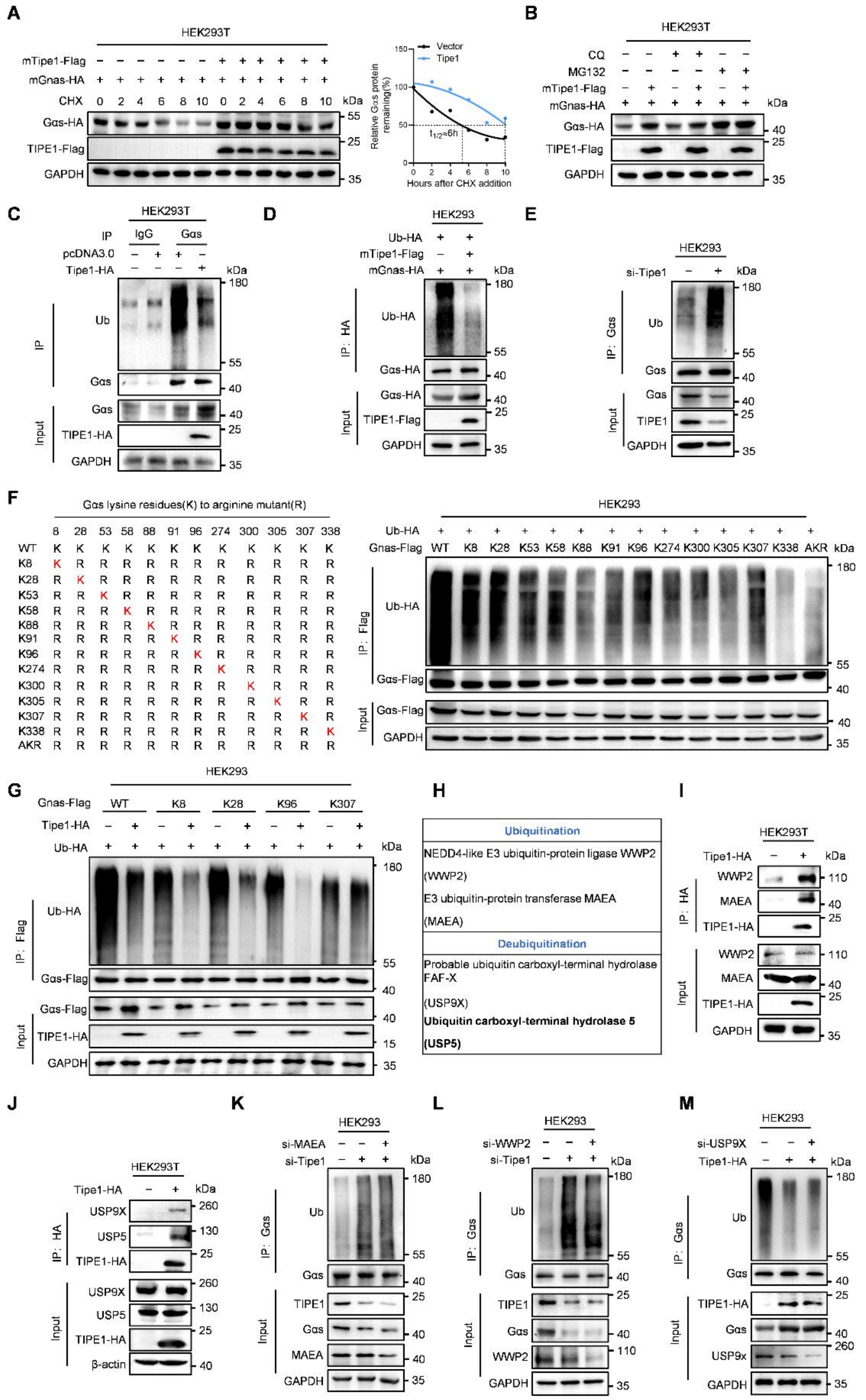

Figure S11. Tipe1 interacts with ubiquitin-associated proteins and deubiquitinates Gαs. A) Cycloheximide (CHX) chase assay. HEK293T cells transfected with mTipe1-Flag or mGnas-HA were treated with CHX (500 μg/ml) for the indicated time points. B) Western blotting analysis of HEK293T cells transfected with mTipe1-Flag or mGnas-HA were treated with Chloroquine (CQ, 10 μM) for 12 h or MG132 (20 μM) for 6 h.

C) Immunoblotting analysis of lysates from HEK293T cells transfected with HA-tagged human Tipe1 (Tipe1-HA), followed by IP with anti-IgG or anti-Gαs. D) Immunoblotting analysis of from HEK293 cells transfected with Flag-tagged Tipe1 (Tipe1-Flag), HA-tagged Gnas and HA-tagged Ub, followed by IP with anti-HA. E) Co-IP of HEK293 cells silenced for Tipe1, followed by IP with anti-Gαs, probed with anti-Ub. F) HEK293T cells were transfected with Flag-tagged Gnas and HA-tagged Ub, followed by Co-IP with anti-Flag, and probed with anti-HA, anti-Flag. G) Co-IP was performed in HEK293 cells overexpressed Tipe1-HA, Gnas-Flag and Ub-HA, followed by IP with anti-HA and anti-Flag. H) List of interacting partners possibly involved in ubiquitination and deubiquitination of Gαs protein. I,J) HEK293T cells were transfected with HA-tagged Tipe1, followed by Co-IP with anti-HA, and probed with anti-MAEA, WWP2, USP9X or USP5. K) HEK293 cells were silenced for MAEA and Tipe1, followed by IP with anti-Gαs, probed with anti-Ub. L) Immunoblotting analysis of lysates from HEK293T cells silenced for WWP2 and Tipe1, followed by IP with anti-Gαs, probed with anti-Ub. M) Co-IP was performed in HEK293 cells silenced for USP9X but overexpressed HA-Tipe1, followed by IP with anti-Gαs, probed with anti-Ub.

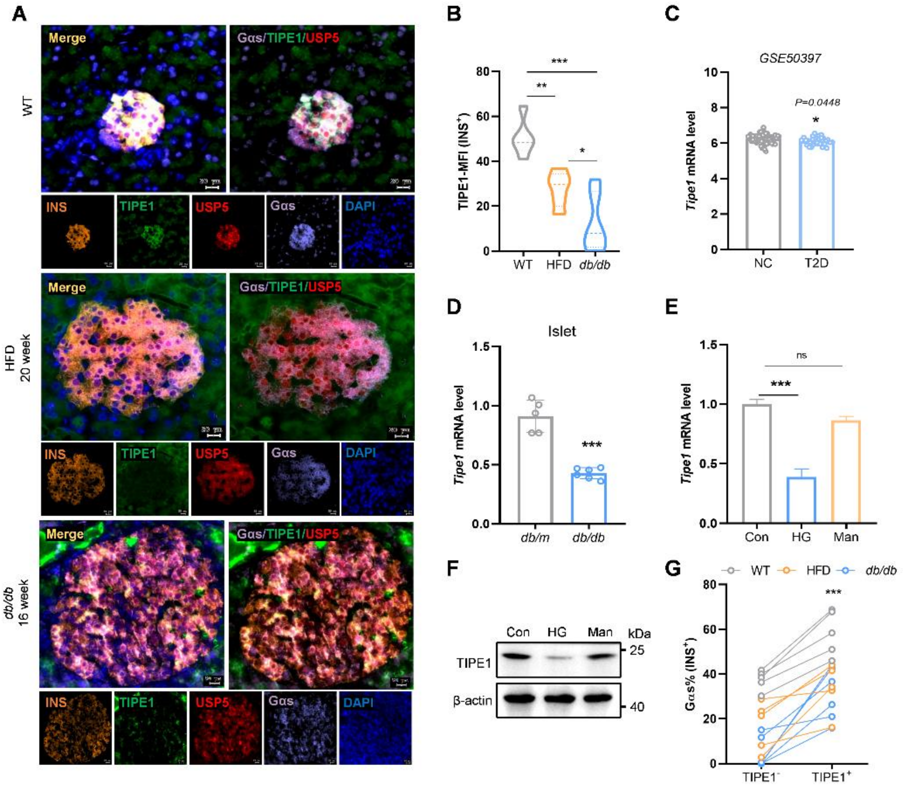

Figure S12. High glucose decreases Tipe1 expression in β cells. A) Representative images for multiplexed immunofluorescence staining (TIPE1, green; Insulin, orange; USP5, red; Gαs, purple) in pancreatic tissues of WT, 20-week-HFD,16-week-*db/db* mice. B) The MFI for the expression of Tipe1 in islet β cells of WT, 20-week-HFD,16-week-*db/db* mice. (n/ WT = 5, n/ HFD = 5, n/ *db/db* = 6). C) A GEO data set analysis. The expression of TIPE1 in ND and T2D (Hba1c＞6; BMI≧24) islets. D) Total RNAs isolated from islets were subjected to qRT-PCR analysis for Tipe1 expression. Tipe1 mRNA was normalized to *β-actin* mRNA. (n = 5 mice/group). E,F) MIN6 cells treated with glucose solution of Con (25 mM), HG (33.3 mM) or Mannose (Man) (33.3 mM) for 48 h, and total RNAs or proteins isolated from MIN6 cells were subjected to qRT-PCR or Western blotting analysis for Tipe1 expression. Tipe1 mRNA was normalized to *β-actin* mRNA. G) The frequency for Gαs positive cells in Tipe1 negative or positive islet β cells in human islet β cells of WT, 20-week-HFD,16-week-*db/db* mice. (n/ WT = 5, n/ HFD = 5, n/ *db/db* = 6).

Data are presented as the mean ± SEM. Data were statistically analyzed by Student's *t* test. **P*<0.05*,* ***P*<0.01, ****P*<0.001, ns indicates no significant difference.
